# Supplementary figures and images for: The role of training variability for model-based and model-free learning of an arbitrary visuomotor mapping
Source: PLoS Comput Biol. 2024 Sep 27;20(9):e1012471. doi: 10.1371/journal.pcbi.1012471 (PMC11463753; doi:10.1371/journal.pcbi.1012471)

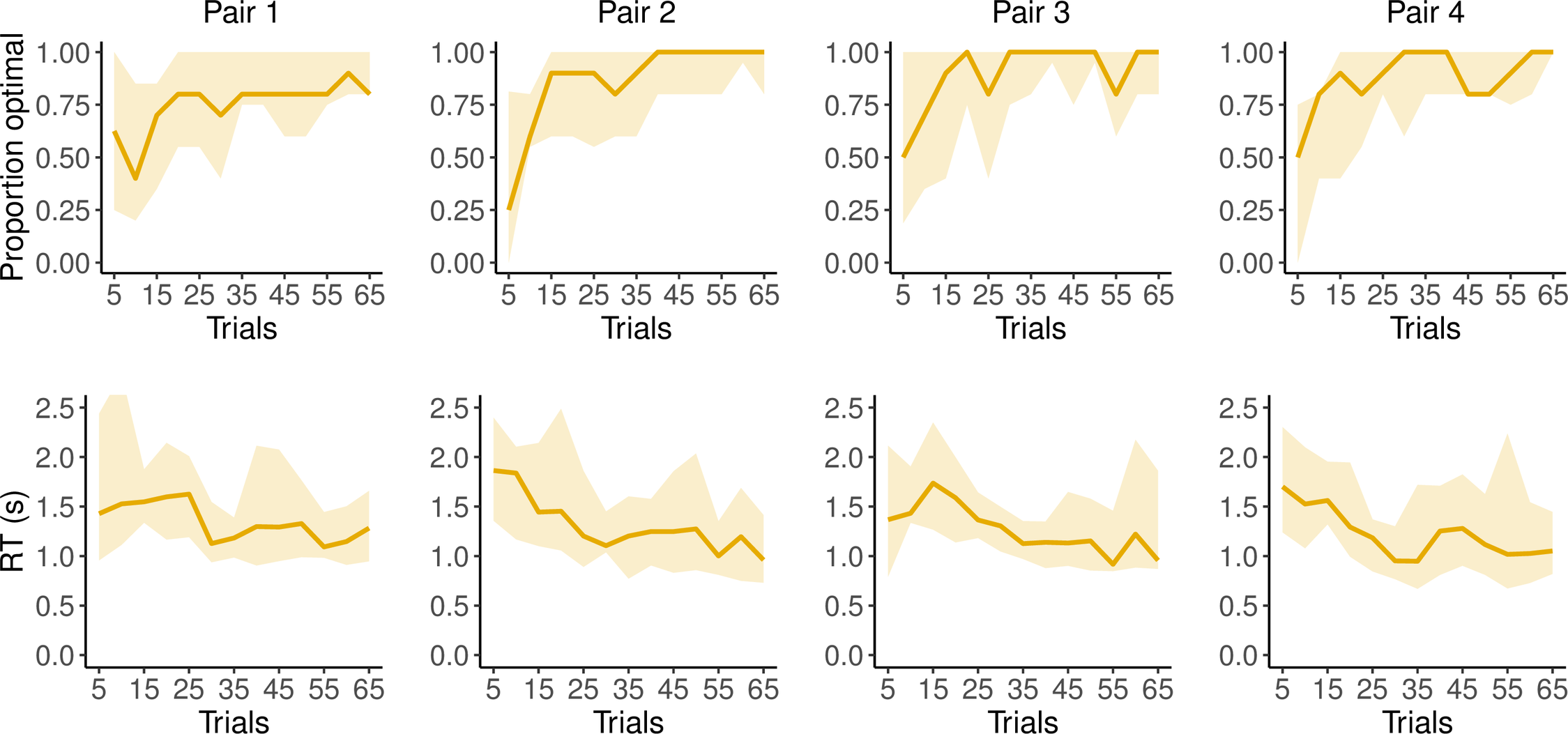

Supplement: S1 Fig — No apparent differences were found in the performance between the different start-target pairs. (TIF) [file pcbi.1012471.s001.tif]

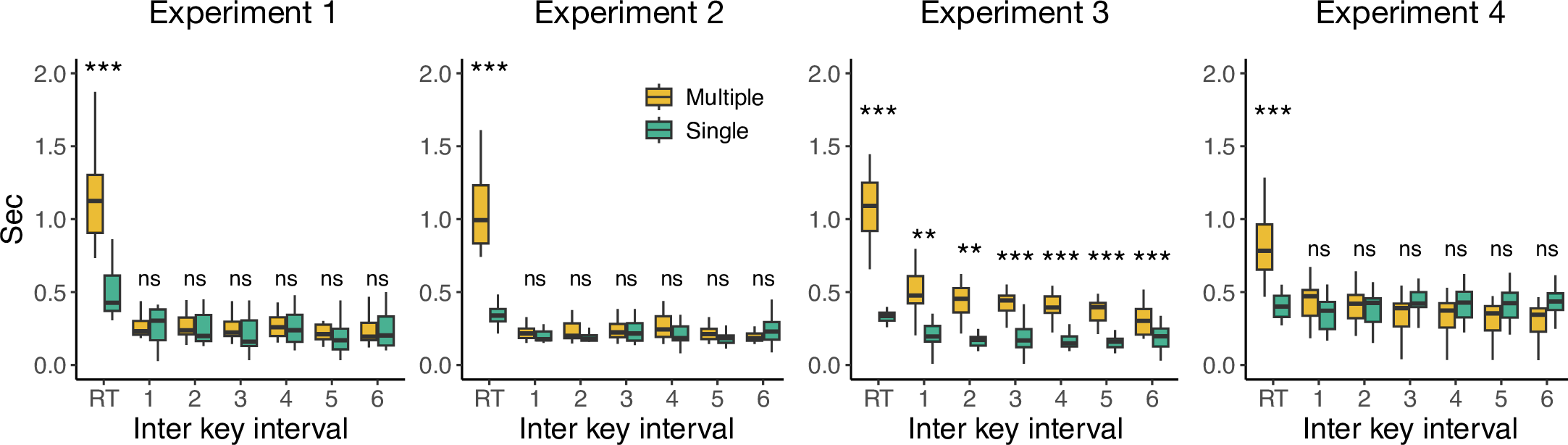

Supplement: S2 Fig — The Single and Multiple groups are shown in gold and green, respectively. (TIF) [file pcbi.1012471.s002.tif]

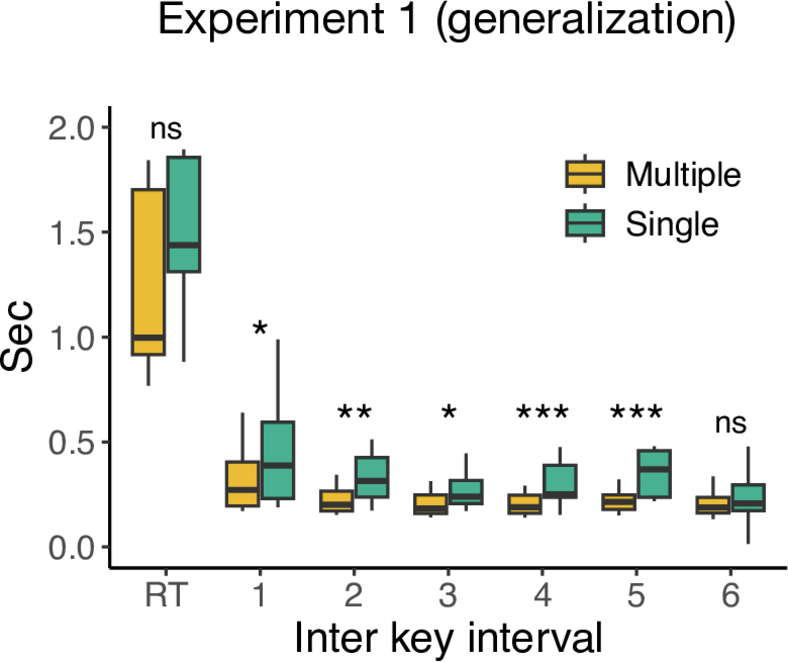

Supplement: S3 Fig — The Single and Multiple groups are shown in gold and green, respectively. (TIF) [file pcbi.1012471.s003.tif]

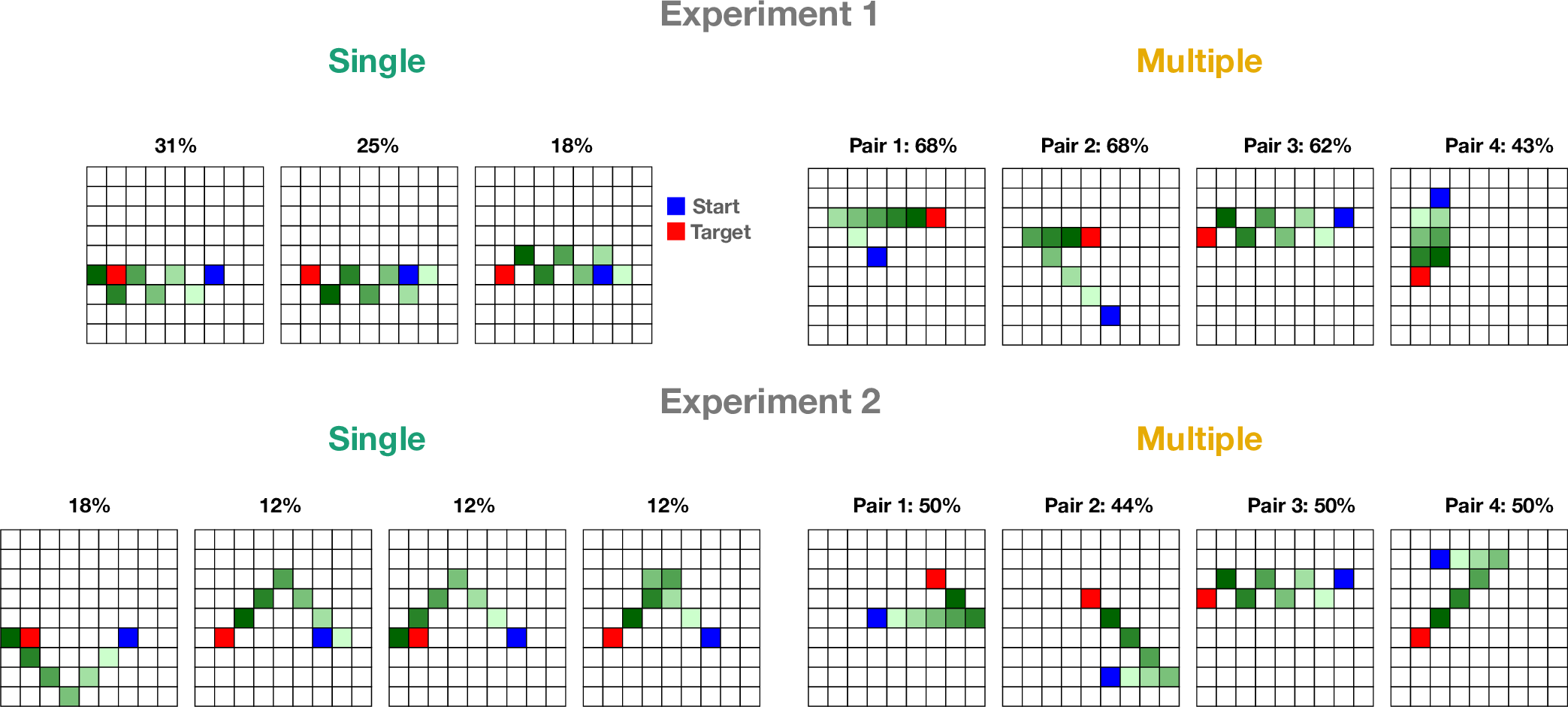

Supplement: S4 Fig — Above each grid, we show the percentage of participants for which the depicted trajectory was the most frequently used during training. We show trajectories that were preferred by at least two subjects. (TIF) [file pcbi.1012471.s004.tif]

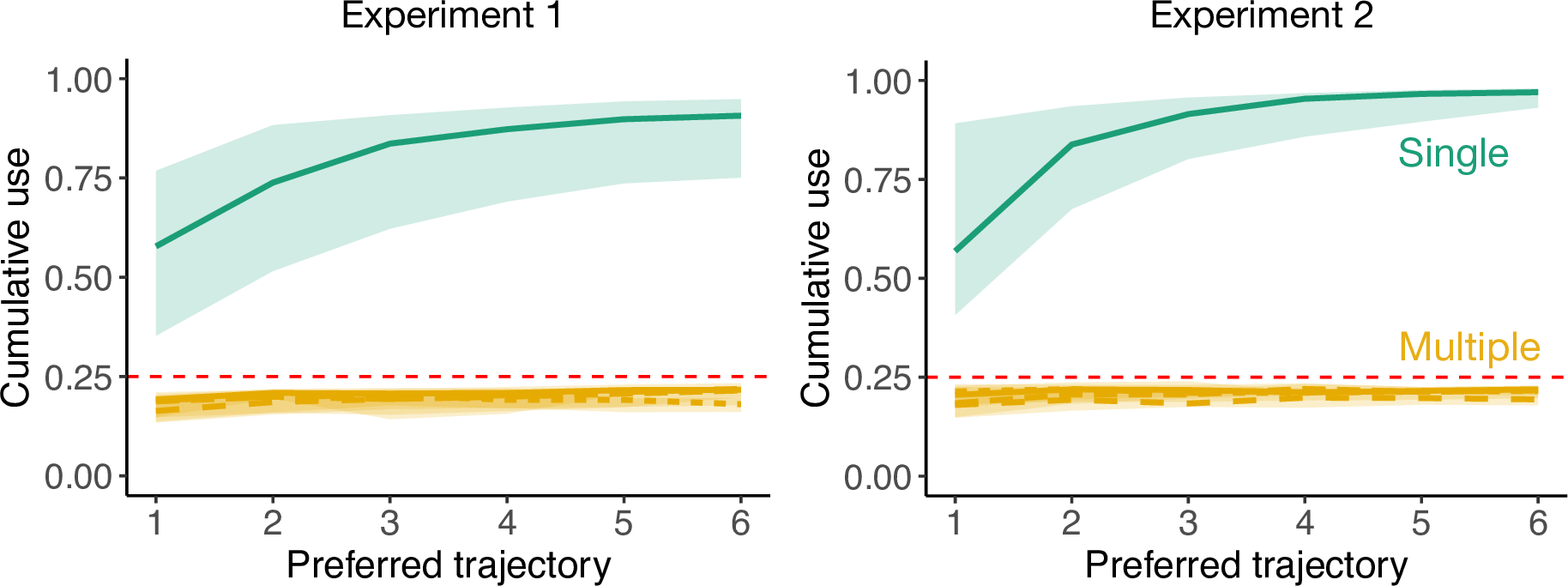

Supplement: S5 Fig — The red line indicates the maximum value for the start-target pairs in the Multiple group (proportion = 0.25), given that each pair only appeared in one quarter of the trials. The different types of gold lines indicate each of the four start-target pairs in the Multiple group. (TIF) [file pcbi.1012471.s005.tif]

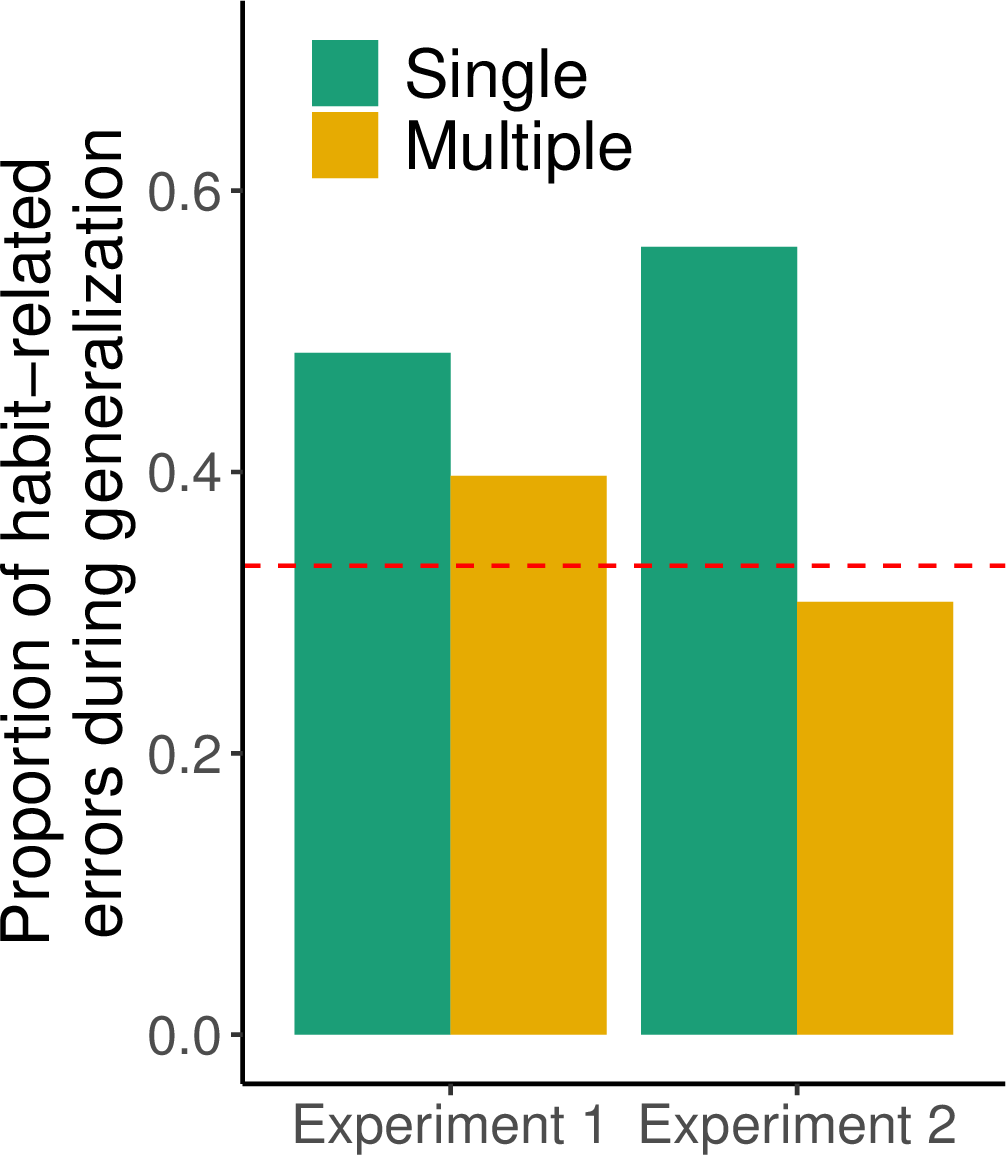

Supplement: S6 Fig — For a given subject, we computed the number of errors during the generalization phase where the first move (the only move in Experiment 2) matched the first move of the most frequently used sequence during their training phase. Given that participants in the Multiple group did not make many errors, we aggregated these values across subjects and divided them by the total number of generalization errors across all participants. We report this value in the Y axis of the plot and the dashed redline denotes chance level. (TIF) [file pcbi.1012471.s006.tif]

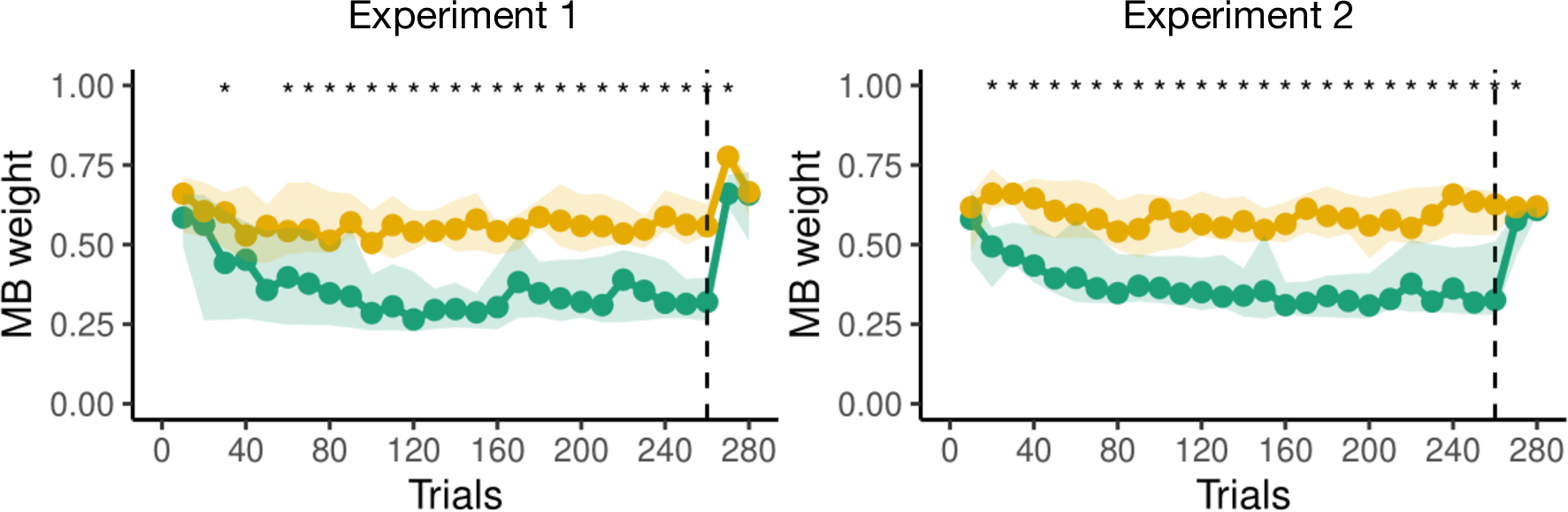

Supplement: S7 Fig — The weights represent a time series of free parameters. A value of 1 reflects fully model-based, while a value of 0 reflects fully model-free. The dashed line demarcates the start of the generalization phase. Since this model has a free parameter per trial, it underperforms the rest of the models when complexity is measured as the parameter counts (Experiment 1: Single: ΔBIC = 2445, Multiple: ΔBIC = 2280; Experiment 2: Single: ΔBIC = 2457, Multiple: ΔBIC = 2214. ΔBIC is the BIC difference in medians with the best model from Tables 1 and 2). However, it provides valuable insights into the dynamics of the weights, which we use to build the arbitration model (AR). (TIF) [file pcbi.1012471.s007.tif]

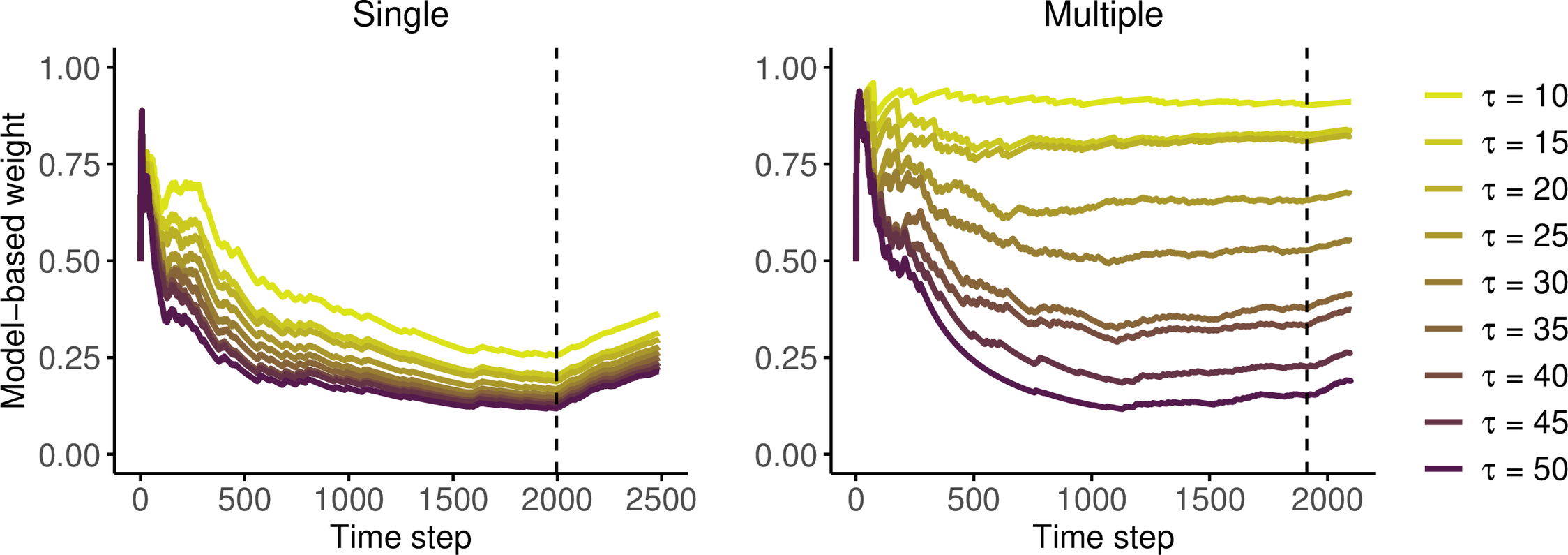

Supplement: S8 Fig — (TIF) [file pcbi.1012471.s008.tif]

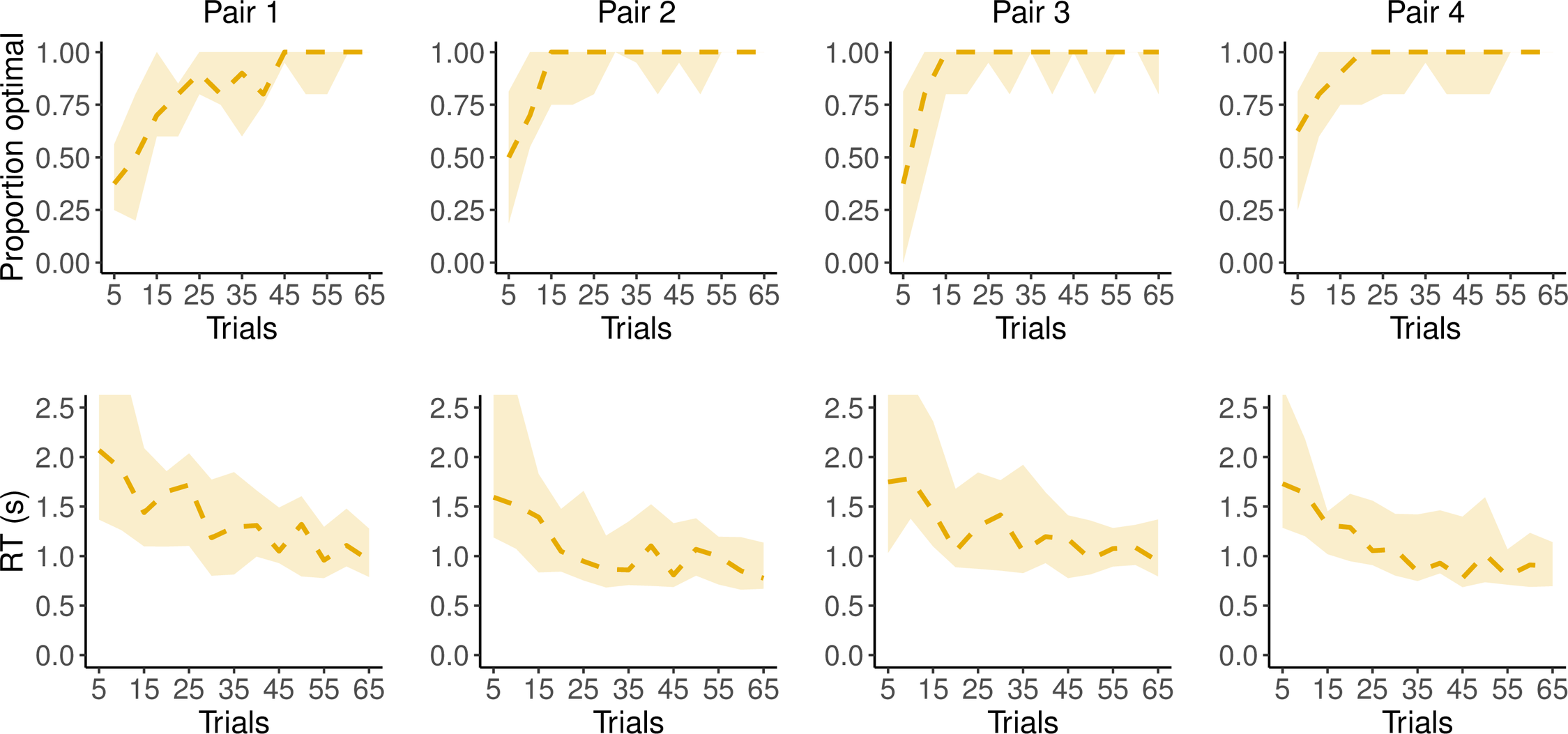

Supplement: S9 Fig — No apparent differences were found in the performance between the different start-target pairs. (TIF) [file pcbi.1012471.s009.tif]

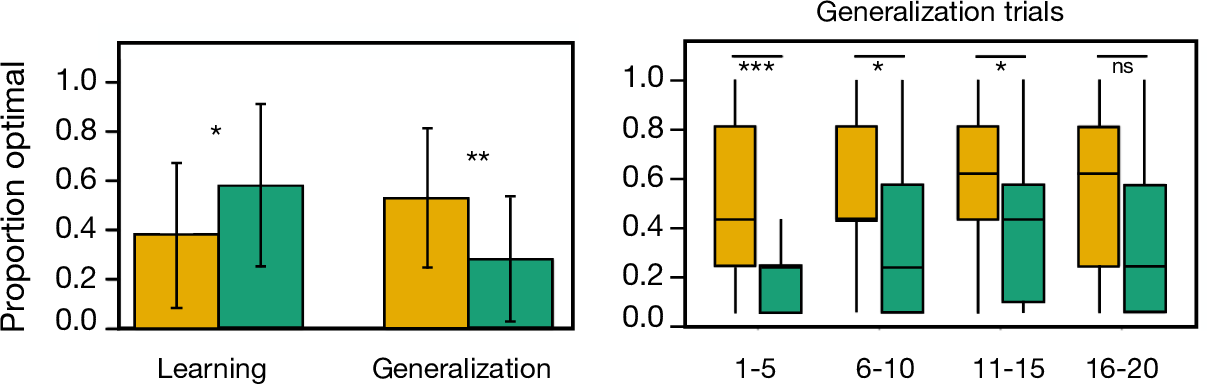

Supplement: S10 Fig — The Single group is represented in green and the Multiple group is represented in gold. (TIF) [file pcbi.1012471.s010.tif]

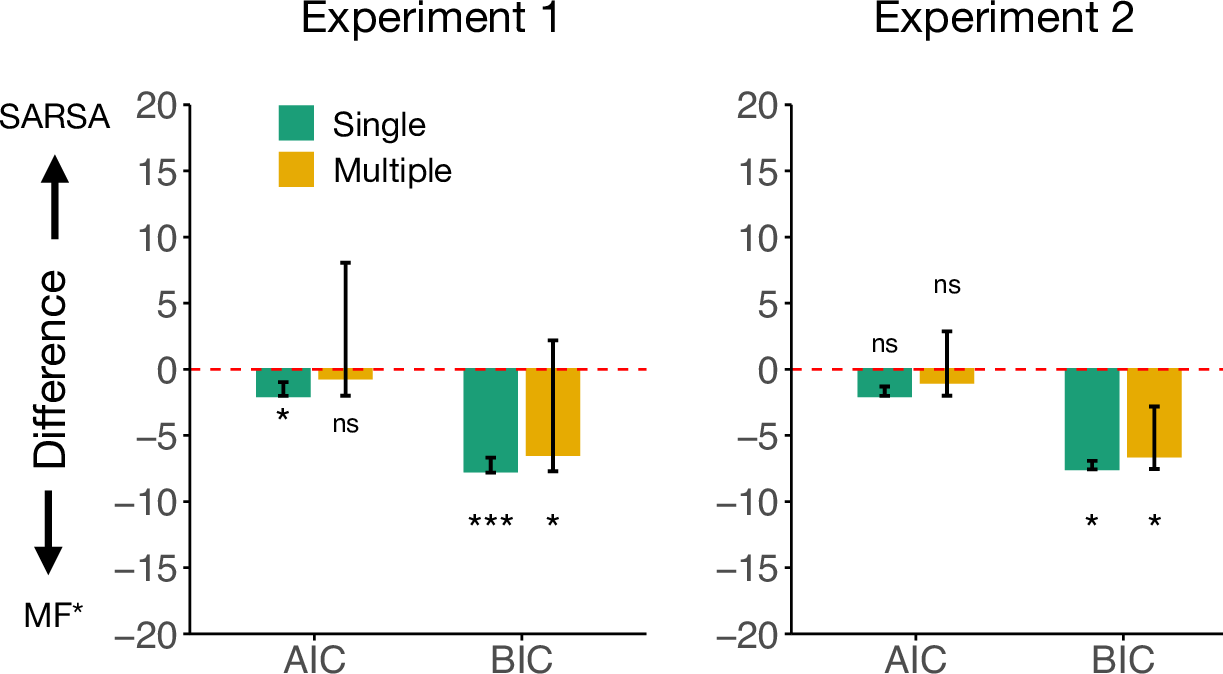

Supplement: S11 Fig — SARSA provides a temporal difference update to state-action values for every start-target pair: Q(s,a)←Q(s,a)+α[r+γQ(s′,a′)−Q(s,a)]. We evaluated the models in the data of Experiment 1 and Experiment 2 using AIC and BIC differences and testing if they were different from zero using the Wilcoxon signed-rank test. We found that our model performed the same or better than SARSA depending on the metric. In particular, the AIC difference was significantly in favor of our model for the Single group (V = 34, p = 0.04), although there was no difference between the models in the Multiple group (V = 77, p = 0.33). According to BIC, which penalizes more strongly the extra parameter γ in SARSA, our model-free algorithm was significantly better for the Single (V = 0, p < 0.001) and the Multiple group (V = 26, p = 0.01). We found similar results for the data on Experiment 2, with no significant difference in performance between the models for the Single (V = 46, p = 0.13) or the Multiple group (V = 67, p = 0.97) according to AIC, but our model outperformed SARSA for both the Single (V = 31, p = 0.02) and the Multiple group (V = 26, p = 0.01) according to BIC. (TIF) [file pcbi.1012471.s011.tif]

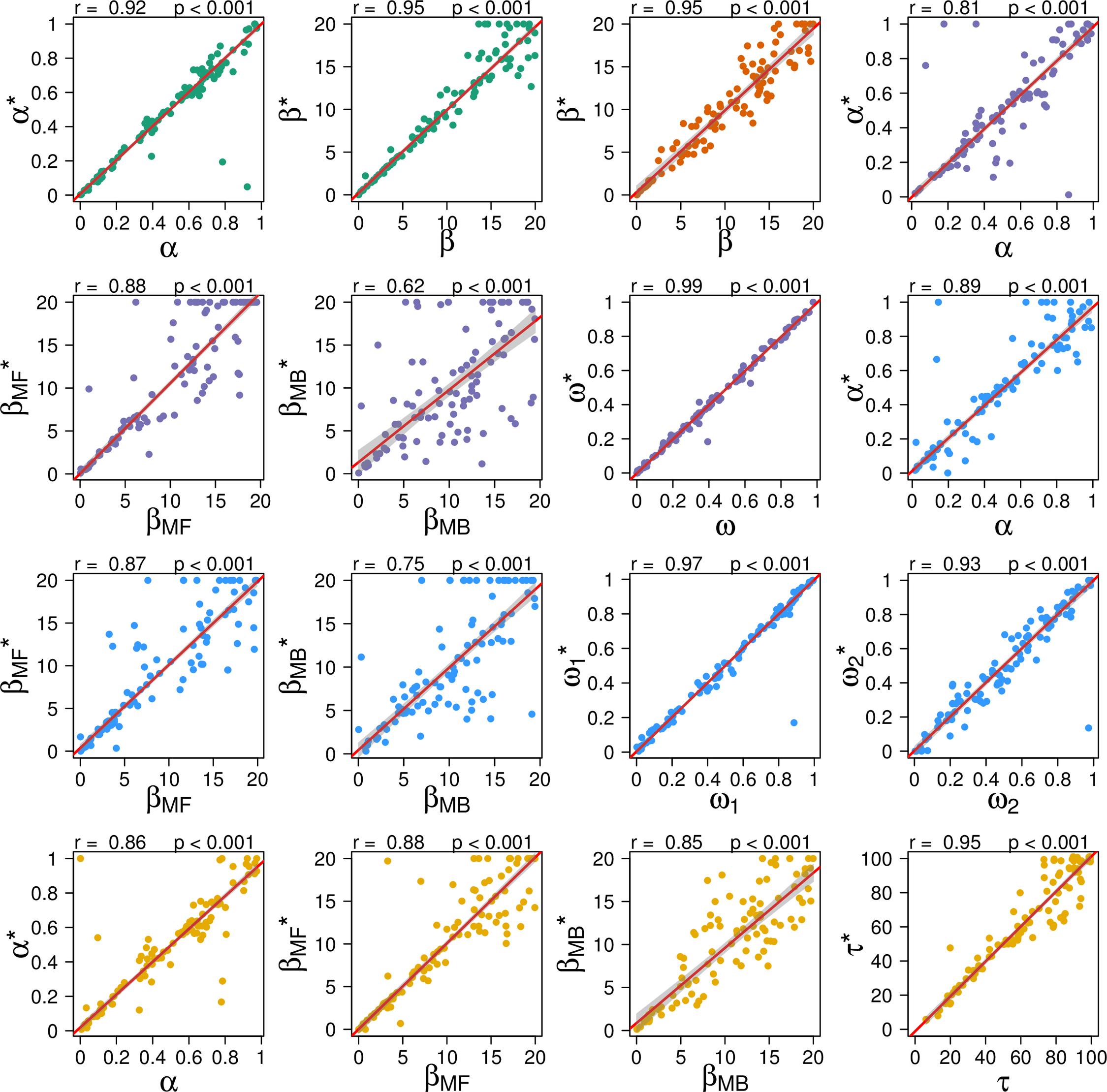

Supplement: S12 Fig — On the x axis is the simulated parameter and on the y axis the recovered parameter. The parameters for each model are indicated with different colors (MF = green, MB = orange, 1W = purple, 2W = blue, AR = gold): α = learning rate, β = inverse temperature, τ = memory window, ω = model-based weight, ω1 = model-based weight in training, ω2 = model-based weight in generalization. Red lines represent the linear fit to the data and the gray shading the 95% confidence interval. On the top of each plot we show the Pearson correlation between the simulated and recovered parameters as well as its associated p value. https://osf.io/zwqj9. (TIF) [file pcbi.1012471.s012.tif]

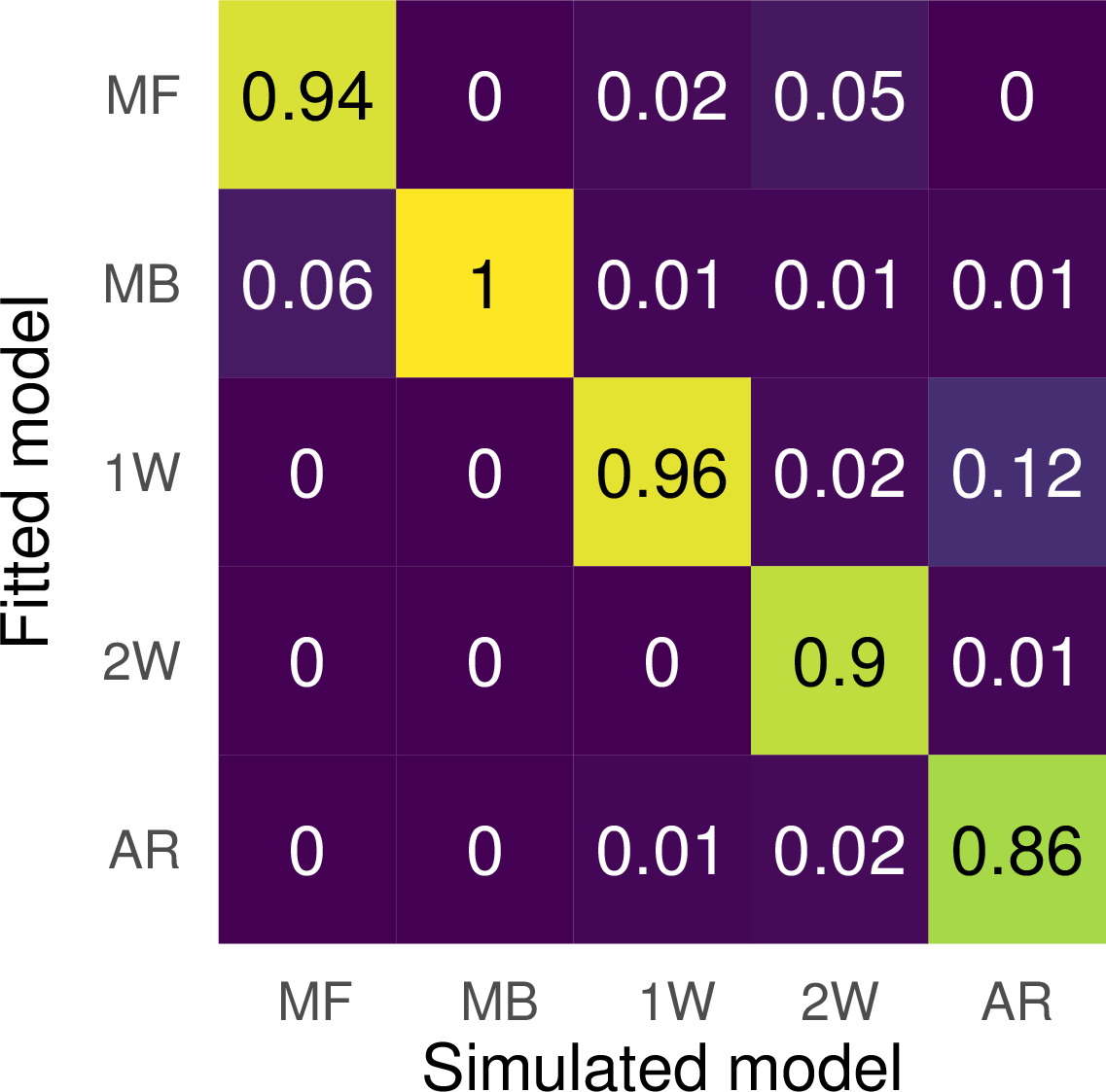

Supplement: S13 Fig — Numbers inside the cells represent the proportion of times that the model in the Y axis best recovered the data generated by the model on the X axis according to BIC. https://osf.io/4xtmv. (TIF) [file pcbi.1012471.s013.tif]
