# Supplementary material for: The role of training variability for model-based and model-free learning of an arbitrary visuomotor mapping
Source: PLoS Comput Biol. 2024 Sep 27;20(9):e1012471. doi: 10.1371/journal.pcbi.1012471 (PMC11463753; doi:10.1371/journal.pcbi.1012471)
Supplement: Attachment — Submitted filename: PLOS_ CompBio_VelazquezDawTaylor_response_letter .pdf [file pcbi.1012471.s014.pdf]

We would like to thank the reviewers for their valuable feedback. We have attempted to address their concerns and made significant modifications to the original text. Here, we list the major changes in the current version of the manuscript:

A) Behavioral: We have incorporated new analyses about the trajectories used by participants to reach the targets; we have provided behavioral measures of model-based computations during the training phase; we have included RTs and inter-key-intervals from Experiment 3 and Experiment 4; we performed analysis of target-specific performance in the Multiple group.

B) Modeling: We have incorporated a hybrid model with an arbitration mechanism instead of the original hybrid model with a time series of free parameters. We have also evaluated two fixed-weight hybrid models; we have included the modeling results of Experiment 3 and Experiment 4.

Together, these modifications corroborate our original findings on the effects of training variability in generalization and the formation of visuomotor mappings. Moreover, they offer deeper insights into the behavioral patterns emerging from the experimental conditions and the cognitive processes underlying participants' performance.

Below we respond to each of the comments. The reviewers' comments are in **bold** font, our reply is in plain font, and specific changes to the text of the manuscript are in *italics*.

<u>Reviewer #1:</u>

This study attempts to bring reinforcement learning into a visuomotor task requiring more in the way of motor learning than the literature's typical bandit tasks do. The internally generated start-goal paradigm with a boat analogy and incongruent action mapping has considerable precedent that needs to be added to here (Fermin 2010/16, Bera 2021, Dundon 2023).

Indeed, our study sought to build on this prior work, which we referenced several times throughout the manuscript (Introduction: Lines 139, 145, Results: Line174, Discussion: Line 1052 Methods: Line 1079, 1088. We do in several places specifically mention the authors' names (e.g., "Fermin and colleagues") to make this more clear.

The matter of generalization such as in many reach and pointing tasks is also an old and familiar one, where the literature on motor control has already demonstrated that training with variety increases the likelihood of generalization. The element with the greatest potential for novelty and impact here is primarily the modeling approach. However, there are fundamental shortcomings in the current modeling despite the potential.

While the variability effect is well known in various more cognitive domains such as language, categorization and problem solving (see Raviv, 2022 for a review), the evidence for such effects in sensorimotor learning tasks is mixed (Berniker et al., 2014;

Braun et al., 2009). We raised this issue in the original manuscript (Lines 874-883 in current version of the manuscript). For example, in a visuomotor adaptation task, Berniker et al. (2014) trained two groups of participants in different workspaces that induced low or high variability in the reaching movements. They found no differences in generalization performance in the novel workspace. In line with these results, several studies in motor adaptation suggest that learning proceeds rather locally (Mattar and Ostry, 2010; Berniker et al., 2013), with a very limited capacity to generalize beyond the trained range. In addition, Throughman and Taylor (2005) found that when the spatial complexity of a force field was increased, which necessarily increases variability, generalization actually narrowed rather than broadened.

On the other hand, Braun et al. (2009) found that participants trained with random rotations, which would average out learning overall but induce variability, were able to adapt faster to subsequent rotations compared to a control group. Their results suggest that training variability can lead to structural learning (i.e., faster adaptation to untrained visuomotor rotations magnitudes). We should note, however, that our lab has followed up on these initial findings from Braun et al. and we found that the variability-induced benefit to generalization was the result of explicit strategies and not implicit adaptation (Bond and Taylor 2017).

Based on these aforementioned studies, we are hesitant to conclude that the effect of training variability in sensorimotor adaptation tasks has been convincingly settled. What's more, it is unclear if the (mixed) findings from sensorimotor adaptation tasks should be extended to grid-world navigation tasks. In fact, McDougle et al. (2022) found that while a patient with severe bilateral hippocampal damage was able to improve her performance in sensorimotor adaptation tasks, she was unable to learn a novel key mapping for grid navigation. This work would suggest that successful performance in the grid navigation task depends on brain regions known to be crucial for spatial navigation and planning (Mattar and Lengyel, 2022), in contrast to the cerebellar-dependant mechanisms involved in motor adaptation (Cullen and Brooks, 2015; Shadmehr et al., 2010; Sokolov et al., 2017). While we ultimately found that variability in training proved critical for generalization of the mapping in grid navigation, we do not think it should have been taken for granted given the mixed findings in sensorimotor learning tasks.

We have incorporated the previous paragraphs into the Discussion section (Lines 874-912). We address the concerns about the computational models below.

In connection with the modeling, a related conceptual issue is that this task is not especially convincing with respect to the authors' argument about "de novo" skill specifically as opposed to recalibration. There are already natural congruent mappings for a spatial layout like this, but the task forces some kind of rotational recalibration or remapping to the incongruent mapping that must be learned. Part of the issue with the

modeling here is that it entirely fails to account for this very plausible alternative interpretation of the task demands. Having said that, taking emphasis away from the "de novo" argument would be relatively harmless in the sense that deemphasizing would not make the results invalid or entirely insignificant if less novel. The focus could be more on the modeling, but again, the modeling is not sufficiently conclusive.

We agree with the reviewer in that there is preexisting knowledge which can make learning in the task not fully *de novo*. For example, participants can have implicit biases to assume that the cursor will move to contiguous locations rather than distant ones when a key is pressed, or that different keys do not move the cursor to the same location.

However, we believe learning in the task is unlikely to be the result of a recalibration process as in motor adaptation paradigms for several reasons. First, although there is a pre-existing mapping between keys and directions specified in the "arrow-keys" of the keyboard, participants did not make their responses using such keys, but instead arbitrary keys (J, K, L) which have a different pre-existing mapping for typing and also a different spatial arrangement than the arrow-keys. The arbitrary association between the keys and the moving directions is what makes this task more *de novo*. Had participants used the arrow-keys, they could have used some rotation rule for each key based on the stamped direction on the key. In fact, the individual with hippocampal damage, mentioned above, was able to perform grid navigation with the arrow keys, but unable to use the less intuitive J-K-L keys arrangement (McDougle et al., 2022).

There can be less intuitive mappings than the one that we used in this task for which learning might be more demanding (for example, Mosier et al., 2005; Velazquez and Taylor, 2023), given the increased degrees of freedom or the number of responses available. However, we believe that the challenge that people face is the same: associating an arbitrary outcome to an action or a set of actions.

In addition, there is evidence that learning in the grid navigation task is substantially different from the one in standard visuomotor adaptation tasks, with the former relying heavily on declarative structures like the hippocampus (McDougle et al., 2022). The hippocampus appears to be a crucial brain region to represent internal models for planning (Behrens, 2018), which could have been fundamental in our task given the sequential and spatial components of it.

Nonetheless, we have taken the reviewer's advice and have deemphasized the use of *de novo* throughout the manuscript, especially in reference to our specific task. While it does appear on occasion it is in general terms. In addition, we have changed the title of the manuscript from: "Learning generalizable visuomotor mappings for de novo skills" to "The role of training variability for model-based and model-free learning of an arbitrary visuomotor mapping"

As this paradigm finds itself no longer in the territory of the bandit tasks that reinforcement learning has proven so effective for, assumptions need to be verified for the specifics of a modeling approach that may not be entirely appropriate for the situation at hand. Though it is an open question, the recently standardized framework of model-free versus model-based versus hybrid (Gläscher 2010, Daw 2011) may actually not be so easily transferable here.

While our task departs from standard bandit tasks, the reinforcement learning framework has also been widely used to model grid navigation (Franklin and Frank, 2018, 2020; Fermin et al., 2010). Indeed, in the original grid sailing task, Fermin et al. (2010) proposed that there was an interplay of model-free and model-based processes across the experiment. However, they did not formally test it. We have taken a further step in this direction by evaluating computational models in the same task as well as their interplay during training and generalization phases. We have proposed fixed weight hybrids as in Glächer et al. (2010) and Daw et al. (2011), as well as an arbitration mechanism aiming to describe how the systems control changes over time.

Without posterior predictive checks and falsification of alternatives (Palminteri 2017), the intended performance of these models has not been properly verified here.

Thank you for the suggestion. In Palminteri (2017), the author mentioned the relevance of simulation of the models prior to fitting the data to evaluate the extent to which they can generate the intended performance. We have now included Figure 4 with the simulations of our models, where we tested the model-free, model-based, and hybrid models for familiar and novel targets, prior to showing the modeling results.

However, in the previous version of this manuscript, we did provide simulations of the models performance to verify that they were identifiable and that the parameter values were reliable (also suggested by Palmiteri (2017)). In particular, we performed parameter and model recovery as have been emphasized in previous work (Wilson and Collins, 2019; Shen and Ma, 2016).

With this, we have incorporated the proposed guidelines suggested in Palminteri (2017), as well as in previous work by Wilson and Collins, 2019.

How comparable are different models when generating predictions against the observed behavior?

In the previous version of this manuscript, we did provide results on how good the models capture the observed behavior. In particular, we used an information theoretic measure of their absolute goodness of fit (Figures 5, 8, 10 and 13; Shen and Ma, 2016). This metric compares the model performance with a theoretical near upper bound for probabilistic models.

Even more concerning is that the explanatory power between specific learning models is extremely limited because of the saturation of learning curves in few trials.

We agree with the reviewer that learning saturates quickly in Experiment 1 and 2 in terms of the optimal arrivals. However, we now include Figure 3 which shows that performance between the groups can differ substantially in terms of which movement paths/trajectories they choose during the training phase (even if both arrive at the target). Particularly, the Multiple group more often chooses options that minimize *only* the true *distance* to the target, which requires relying on the key-mapping for planning. This distinction, likely generates the difference in the model-based weights between the groups observed in the training phase (Figures 5 and 8)

In addition, we have now included the modeling results of Experiment 4 where, given the stochasticity of the task, participants learned more slowly, only reaching near ceiling performance by the end of the training phase. The design of Experiment 4 generated a richer data set in terms of the variability of participants' responses and also provided converging evidence that, as in Experiment 1 and 2, variability in training leads to better generalization; with the distinction that variability in Experiment 4 was induced by a stochastic mapping.

However, we do believe that tasks that resemble the complexity of the mappings that humans learn in their life, and where learning does not saturate as quickly, should be studied. Currently, our group is working in this direction (Velazquez-Vargas and Taylor, 2023). We have highlighted this point in the discussion, in Line 1061:

> *"However, it is important to note that in contrast to the complexity of the mappings that humans learn in their lives, the mapping that we tested in our studies was relatively simple. Therefore there is a need to study mappings with greater complexity. In a recent work (Velazquez-Vargas and Taylor, 2023), we provided a step in this direction by asking participants to navigate in the grid using a mapping based on the less intuitive rule of the 'Knight' from chess. This and similar settings can allow the study of the processes, such as learning and planning, involved in the acquisition of complex skills"*

As suggested throughout this review, there is plenty of grounds for skepticism about whether these models are even remotely close to how humans actually solve the task (which, as the authors correctly point out, also differs from one person to another with presumably large individual differences here).

While it is unclear if this is what participants really do in the task, our models generate testable predictions that resemble the distinct patterns of generalization observed in our groups. It is worth noting that these classes of model-free and model-based processes suggested in the original grid sailing task (Fermin et al., 2010, 2016). In the Discussion we have pointed out that further variations of the models can be tested in future work,

which can improve the models' performance and make them more biologically plausible. For example, the models could benefit from the incorporation of a persistence component in responses (Daw, 2011; Miller et al., 2019), by varying the planning search used in the model-based algorithm to incorporate resource rational constraints (Mattar and Lengyel, 2022), or even by including mechanisms that do not assume model-based planning at all (Colas et al., 2022). See Lines 986-1016, in the Discussion section.

Beginning with the model-free model, it seems arbitrary in multiple respects and not especially optimal here. Why not begin with something closer to standard temporal-difference learning (Sutton 1998)?

We agree with the reviewer in that temporal-difference is a more standard algorithm than the model-free algorithm we used. Although we did not include it in the first version of this manuscript, we had initially tested SARSA (Sutton and Barto, 1998) which provides a temporal difference update to state-action pairs: $Q(s,a) \leftarrow Q(s,a)+\alpha[r+\gamma Q(s',a')-Q(s,a)]$. This model has the additional term $\gamma Q(s',a')$ compared to our current model-free algorithm. Here, we show the evaluation of both models with the data of Experiment 1 (A) and Experiment 2 (B), using AIC and BIC differences and testing if they were different from zero using the Wilcoxon ranked-test test. We found that our model performed the same or better than SARSA depending on the metric. In particular, for Experiment 1, the AIC difference was significantly in favor of our model for the Single group ($V = 34$, $p = 0.04$), although there was no difference between the models in the Multiple group ($V = 77$, $p = 0.33$). According to BIC, which penalizes more strongly the extra parameter γ in SARSA, our model-free algorithm was significantly better for both the Single ($V = 0$, $p < 0.001$) and the Multiple group ($V = 26$, $p = 0.01$). We found similar results for the data on Experiment 2, with no significant difference in performance between the models for the Single ($V = 46$, $p = 0.13$) or the Multiple group ($V = 67$, $p = 0.97$) according to AIC, but our model outperformed SARSA for both the Single ($V = 31$, $p = 0.02$) and the Multiple group ($V = 26$, $p = 0.01$) according to BIC. These results are summarized now in Figure S10:

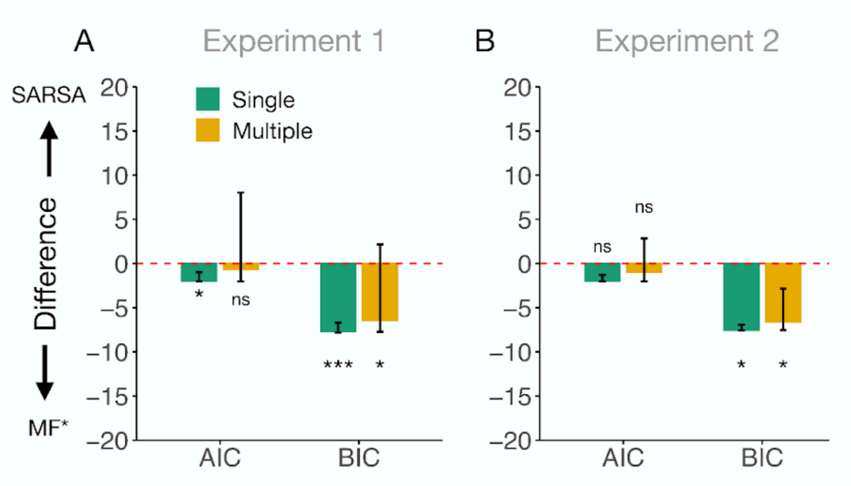

MF* represents our model-free algorithm.

These results suggest that the incorporation of the term γQ(s′,a′) in SARSA does not bring significant improvement in the fit to the data – with the additional parameter γ increasing the complexity of the model.

We believe that the inclusion of an additional model-free algorithm with similar or worse performance would not significantly affect the results of the manuscript. However, we now include the previous analysis to the supplementary material to justify the selection of our model (Lines 1232-1234).

Having said that, picking the exact setup for a primarily model-free alternative (for example, defining “states”, “actions”, and “rewards” here) would be nontrivial in this novel situation. As compared with a two-armed bandit, this is a very challenging problem for a more complex task. For example, can the authors justify states as the grid, actions as generic choices, or reward as a distance reduction rather than the standard reward of the actual goal? The list of other possibilities is a long one. Nobody has the answers to questions like these yet, and it would be naïve to assume that one solution arrived at somewhat arbitrarily is going to be a viable final solution. Multiple possibilities can be explored, and coexistence of different algorithms is of course possible as well with a much larger space than that of bandit tasks.

We appreciate the reviewer's insight regarding the challenge of defining elements in the reinforcement learning framework (states, actions or rewards), a topic addressed in previous works (Wilson et al., 2014; Schuck et al., 2016; Schuck et al., 2018). Below, we provide our rationale for defining these elements in our experiments:

States: In reinforcement learning, states can be broadly defined as "the collection of information relevant to a given decision" (Schuck et al., 2016). Wilson et al. (2014) highlight that states can range from a simple binary condition, such as a light being on or off, to the complex configurations of pieces in chess. In our model-free algorithm, a state was defined by the cursor and target locations on the grid (in absolute coordinates), with decisions made by comparing action values for each state. This approach aligns with previous theoretical work by Franklin and Frank (2018) and their subsequent application to human data (Franklin and Frank, 2020; Equation 4). In more complex tasks such as board games or video games, it is conceivable that states can also increase in complexity or be partially observable (Wilson et al., 2014; Heald et al., 2021), including obstacles or multiple contexts. However, our task was relatively simple compared to those scenarios with no obstacles in the grid or hidden contexts.

In addition, while in the original grid navigation task from Fermin et al. (2010, 2016) the authors did not address the definition of state explicitly, it is implied from the definition of the action-mapping as a an internal model of “state transitions”, that states also refer to the locations of the grid.

Whether our implementation of state in our model-free agent is accurate is beyond the scope of our current manuscript and can be addressed in future work.

Actions: Similar to previous work (Franklin and Frand, 2020; Equations 6 and 7), we have represented actions in our models as individual key presses. This simplification may overlook scenarios where sequences of key presses are chunked into single action (Bera et al., 2022). It is conceivable that participants initially choose individual key presses, but as the experiment progresses, they may select entire sequences. Our current model does not account for this or similar scenarios where actions can be defined differently. Again, a possibility for future work and beyond the scope of this paper, which is already lengthy.

Rewards: In our task, rewards depend on whether the cursor moves closer or away to the target on every move using the chessboard distance. We made this assumption to aid learning in our model-free algorithms as explicit rewards (emoji faces at the end of the trial) were relatively rare compared to the number of moves in the task. We have made reference to this point in Lines 1236-1239. We found that if we only used the endpoint feedback as a teaching signal for the models, learning proceeded considerably slow (which contrasts with the steep learning curves). Indeed, when we implemented a variation of the SARSA algorithm that relies on sparse rewards, we found that it performs remarkably worse than its counterpart using distance reduction as reward. In particular:

Experiment 1: mean Δ BIC = -911 for the Single group and mean Δ BIC = -1644 for the Multiple group.

Experiment 2: mean Δ BIC = -924 for the Single group and mean Δ BIC = -1541 for the Multiple group).

A sparse-reward version of our model-free algorithm would have performed considerably worse, as, unlike SARSA, it does not propagate back the rewards.

The possibilities are not just about learning strategies either, especially because the learning curves saturate so quickly. Importantly here, the majority of the cognitive work concerns action planning and execution (e.g., route planning versus heuristic planning in Dundon 2023). Training a typical simulated model-free agent to do this task would not share all of the circumstances of the human doing the task, such as an incongruent action mapping (van Opheusden 2019). Humans learn the task rather quickly with the aid of its natural familiarity, which again allows for a recalibration argument contrary to the "de novo" argument. It seems highly implausible that a person looking at a boat and target here would be completely model-free the way a classic RL agent is before undergoing many trials in simulation.

We agree with the reviewer that “*de novo*” may be too strong in our case and have deemphasized it in the revised manuscript. However, we still feel that it shouldn’t be considered simple recalibration either since participants are unlikely to have any previously learned association between keys “J-K-L” and movement of a cursor. Also, it is in some intermediate zone where the participants likely bring inductive biases to the task, making it not completely *de novo* and afford faster learning than a completely model-free agent would learn.

While it is true that the task would be considerably challenging for a model-free agent relying entirely on sparse rewards, the model-free algorithm can speed up the process significantly by relying on the distance reduction as reward. This reward structure allowed us to simulate performance that is similar to what we observed in our experiments.

The following figure shows the proportion of optimal arrivals in 100 simulations of our model-free algorithm exposed to the same start-targets as the Multiple group (260 training trials). We used a learning rate of $\alpha = 0.75$ and an inverse temperature parameter $\beta = 5$, for the simulations.

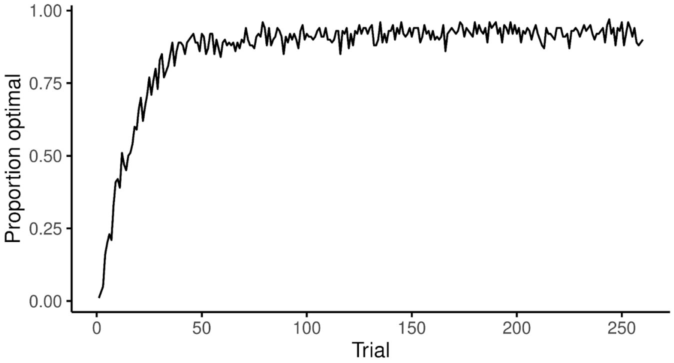

It is important to note that both model-free and model-based algorithms can reach ceiling performance in the task as can be now observed in the simulations of Figure 4. However, a key difference between them is which choices they make to get to the target. While the model-free algorithm prefers options that minimize the chessboard distance, the model-based algorithm, relying on the estimates from Breadth First Search, also finds solutions that minimize the true, model-based distance.

As far as the model-based model, the essence of the perspective problem is the same as that raised with model-free. A powerful tree-search algorithm like breadth-first search of course can ultimately solve the task with performance comparable to that of a human, but this does not mean that brains are solving the task in the same way (Navarro 2019). Why this particular algorithm as well as this sort of tree-search class? And what about the aforementioned constraints of this task with spatial navigation, incongruent action mapping, etc.?

We appreciate the insight from the reviewer. Although Breadth First Search (BFS) is suitable to solve deterministic tasks like ours, recent work suggests that algorithms that incorporate resource rational constraints or heuristics are more likely to be implemented by the brain (Mattar and Lengyel, 2022). We decided to implement BFS given its uniform structure which allows it to explore all paths that lead to the target (even if not immediately obvious). This was particularly important given that participants often chose moves that took the cursor away from the target, which would have been harder to detect in search algorithms that use as a heuristic some unconstrained metric (e.g., the chessboard distance or the Euclidean distance). However, our implementation of BFS assumes that participants can plan with infinite depth, which is an unrealistic assumption. Future variations of this model can include limits in the planning depth known as “lookahead” which can be more biologically plausible. We now address this point as an important limitation of our work in Lines 996-1009.

Our initial goal in terms of computational modeling, was to select algorithms that could generate the distinct generalization patterns observed in our experimental groups. The reinforcement learning framework with its broad distinction between model-free and model-based algorithms allows to formalize these patterns effectively.

Although it remains an open question which planning algorithms people actually use, our experimental conditions were not specifically designed to dissociate between them. Consequently, we did not have specific hypotheses regarding which planning patterns to expect.

As the separate models are not telling either side of a truly complete story here, the hybrid model is also uninterpretable. But why are the authors opting for hundreds of free parameters for weight parameters by trial in the hybrid model? What can be concluded from a model comparison with 1-parameter models versus hundreds? (The use of two training and generalization weights for the simulations is also problematic instead because they are too coarse, but at least the parameter count is reduced.) As opposed to a generative model with arbitration (Lee 2014), for example, this also leaves the model incomplete in its attempted explanation of the actual cognitive process, which could in principle be achieved with only a few parameters but is again challenged by limited data for a saturated learning curve. Having said that, it could also be the case that the most standard dichotomy of model-free versus model-based is not really appropriate at all here in the first place.

We appreciate the comment of the reviewer. We have two major modifications to the paper to address this point.

First, we removed the hybrid model with the time series of free parameters from the main text of the manuscript and moved to the Supplementary material (Figure S2). Our reason for wishing to keep the hybrid model with the time series of free

parameters is because it remarkably shows a relatively smooth and consistent function, even though the model is completely unconstrained. This suggests to us that the performance of the model couldn't be solely attributed to overfitting and we had used the log marginal likelihood to attempt to account for the number of free parameters. Regardless, we now leverage an arbitration mechanism that aims to capture the dynamics of the model-based weight observed in the unconstrained model. The arbitration in this model relies on the familiarity of states and predicts differences in the model-based weight for the Single and Multiple groups (Figure S3). It can qualitatively capture the decrease in the model-based weight over time in the Single group, as well as the consistently higher model-based weight for the Multiple group. Although it did not provide the best description of the data at the population level (Figure 5, 8, 10 and 13), it did provide the best description of the data for several subjects in Experiments 1, 2 and 4 (Table 1, 2 and 4).

Second, rather than assuming that the model-based weight changes through time, we implemented a 1-weight hybrid model, where the weight remains the same across the experiment. Similarly, we implemented a 2-weight hybrid model, which has different weights for the training and generalization phases. The 2-weight hybrid model provided the best description of our data in all experiments at the population level.

As an alternative to pure model-free, pure model-based, and hybrid models, one could plausibly consider a more singular modeling approach that is not fully model-based but does use a cognitive map for generalization of model-free learning signals and hippocampal experience replay (Liu 2021, Colas 2022). In this case, a two-dimensional grid has natural overlap in directions of movement, paths, regions, and rotational symmetry for possible dimensions to generalize across (from the actual sequence taken to hypothetical variations of that sequence elsewhere on the grid). From this perspective, it seems obvious that humans are not going to be fully model-free in this familiar spatial-navigation task, but they also do not need to be fully model-based in an exhaustively optimal Bayesian sense in order to perform well at this task very quickly. Flexible generalization of model-free information such as reward prediction errors could be more involved in the modeling here to help simulated agents learn as quickly as humans do.

We appreciate the valuable references provided by the reviewer. We agree that it is possible to generalize using only a model-free algorithm as suggested in the work by Colas et al. (2022). However, in our studies, we found behavioral evidence that strongly suggests that participants relied on an internal model for planning. In particular, when contrasting the generalization performance between Experiment 1 and Experiment 2 (Figure 7), we found that RTs were significantly higher when the target locations were distant (seven moves away) as compared to when they were proximal (one move away) for both the Single and Multiple groups. Moreover, the performance in terms of optimal

arrivals between the Multiple group of Experiment 1 and the Multiple group of Experiment 2, was statistically at the same level, with the only difference that participants took longer to respond to distant targets in the latter. We interpret these results as evidence that participants were planning to arrive at the novel targets in generalization, and that this process was computationally more demanding when target locations were distant. While the Single group is most likely less efficient in performing these computations, they still show evidence of doing so (Lines 913-929 in the discussion).

Indeed, in another experiment using a slightly more complex mapping, we investigated how RTs changed with varying degrees of planning horizon. We found that they systematically increased as the target was further moved away:

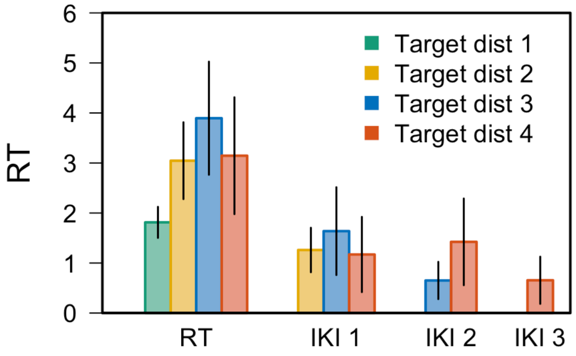

In addition, we performed a new analysis of responses during the training phase that suggest a more frequent use of knowledge about the state transitions in the Multiple group (Figure 3). In particular, participants in the Multiple group were more likely to choose moves that took the cursor in a direction that was away from the target while reducing the actual distance to it (in terms of the number of moves). We believe such choices are more likely to arise from a process that considers the final outcome of a series of actions than from a model that is unable to plan.

With the modeling being underdeveloped and unverified, the manuscript and the work as a whole just feels rather incomplete. The model comparison with a mere three models of questionable validity leaves the interpretation of interesting (but not entirely novel) behavioral results inconclusive. On a related note, where is the rest of the modeling for Experiments 3 and 4? For example, what can be said of the decay of forgetting in Experiment 3? And Experiment 4 could yield distinct information about the actual learning process. There is still much to explore here. Further exploration could realize more of this work's potential despite the tone of this review that suggests not yet publishing as it is currently.

We appreciate the detailed and thoughtful comments of the reviewer. As noted above, we opted for the model-free and model-based approach because that was the approach put forth by Fermin and colleagues. During the development of this project, we explored a number of models, especially model-free processes that could afford apparent

generalization but our behavioral findings suggested that a model-based algorithm was necessary. We feel that the now classic approach of model-free and model-based processes captures the behavior in a general and straightforward way.

Per the reviewer's suggestion, we have incorporated the modeling results of Experiment 3 and 4. In summary, In Experiment 3 we found that the model based weights in the two generalization phases (before and after the prolonged period of no variability) were not statistically different. These results support our behavioral findings that a brief period of training variability can have long term benefits in generalization. In addition, in Experiment 4, we found that the model-based weights between the Single and Multiple group are not significantly different in the training or generalization phase, corroborating the behavioral findings that training variability in the form of stochastic training can allow to flexibly switch to model-based computations in the face of novel targets.

__**Reviewer #2:**__

I think this is a good paper and a good fit for this journal. Velázquez-Vargas and colleagues performed a series of 4 experiments in a navigation task designed to encourage participants to learn de-novo motor mappings. By varying the training schedule, they were able to show either a presence or absence of generalization, and buttressed their findings with computational modeling.

We thank the reviewer for the positive comments.

I have two primary concerns about the work. One, while I liked the modeling, I would have liked to see generative simulations as to whether their hybrid model is able to capture the qualitative patterns in the behavioral data.

We have now incorporated generative simulations of the models in Figure 4. We show that the model-free, model-based, and single weight hybrid models are able to generate ceiling performance for familiar targets. However, for novel targets, the model-free algorithm generates chance level performance, whereas the model-based algorithm predicts full generalization. In contrast, the hybrid model generates a wide range generalization performance which varies depending on its weight, more closely resembling the human data.

Second, I was unconvinced by experiment 3 and am not sure what value it has in the paper. In experiment 3, the authors seem to be interpreting a null result. There is no difference in generalization prior to or following the Single block, and the explanation proffered by the author's is that "early exposure to variability can allow people to learn the visuomotor mappings and that they can recall it in the future if novel goals appear". My complaint about this is what is the null hypothesis? Subjects are continually practicing on a task that uses the learned visuomotor mappings during the Single block, and I don't think you can make any claims about what is happening during the

single block without a change in behavior. As such, I think the proper null hypothesis should be that there is no change in behavior during the single block, and that is what is observed.

We saw two possibilities, which are outlined in the main text on Lines 611-621: 1) forcing participants to initially learn the mapping may afford generalization even after a long period of repetitive training at a single target (the null hypothesis as the reviewer points out) or 2) participants could eventually forget the mapping if they were only required to repeat movements to one target location. This is like if a budding pianist first learned their scales ("mapping") but then practiced only a single melody ("sequence", for an extended period, they might forget the meaning of the piano keys in relation to the scale degrees – the senior author has experienced this firsthand. In addition, in Experiments 1 and 2, it was unclear if the participants in the Single group ever learned the mapping since the task didn't necessarily demand it. Instead, they could simply memorize a sequence of responses and repeat it throughout the entire experiment. With these two points in mind, we designed Experiment 3 to test whether repetitive training to a single target would afford generalization even after a long period of training or it would cause forgetting of (or interference with) the full mapping, while also ensuring that the participants had learned the mapping in the first place.

While the outcome of Experiment 3 supported this null hypothesis, we did find a change in behavior in terms of RTs, which is now presented in Figure 10. In particular, RTs significantly increased from the end of the Single block to the beginning of the generalization phase. Such an increase was also present in the Single group of Experiments 1 and 2, but it was not accompanied by successful generalization like in Experiment 3. We interpret these results as participants switching from model-free to model control successfully, which is also supported by the weights of the hybrid model now presented in Figure 11C. In particular, the model-base weights significantly change from near zero in the Single block to around 0.8 in the subsequent generalization phase.

Other comments:

In experiment 1, I didn't follow the explanation of why RT increased in the Single group.

We interpreted the increase in RTs from training to generalization in the Single group as switching to model-based control. Even though the Single group was not as good as the Multiple group in arriving at the novel targets, they were able to generalize to some extent. In addition, when comparing the results from Experiment 1 and Experiment 2 in the generalization phase, we found that the Single group (similar to the Multiple group) had higher RTs for the distant targets compared to the proximal targets. These results suggest that, during the generalization phase, participants were engaged in planning computations, which are more demanding for distant goals, and that the increase in RTs from training to generalization were not merely due to the novelty of the targets.

In experiment 4, I wonder if your metric is not as sensitive as it could be. Instead of proportion arrivals, have you looked at their responses on a step-by-step basis for

optimal responses (for example, if the target is one-step to the right, then a response "K" should be optimal, regardless of whether the cursor moves to right)? That might give you more statistical power.

We agree with the reviewer's intuition that number of arrivals appears to be a coarse measure of performance. However, the problem with using step-by-step optimal responses is that optimal moves are more likely to occur by chance than an arrival to the target. The reason for this is that the majority of locations on the grid have more than one optimal move from the three available options. For example, at the starting point of the Single group, any of the three keys reduces the distance to the target. In contrast, arriving at the target typically reflects a deliberate strategy, such as executing a correct sequence of n moves, which is less likely to occur by chance. Indeed, the number of possible trajectories that do not lead to the target is much larger than the ones that arrive at it.

When we examine the learning curves using the number of optimal moves, we find that it is less sensitive to capture the performance curve compared to the number of arrivals to the target. This can be observed by the subtle change from the beginning to the end of the experiment:

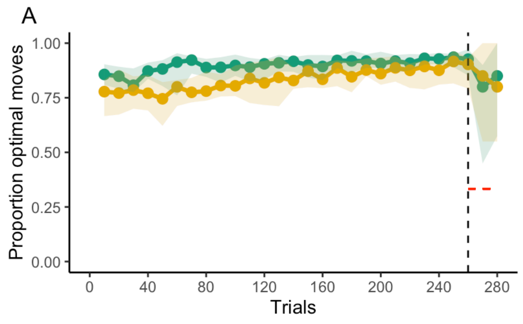

As for the modeling, I really liked the approach. I was slightly confused by omega and how it was determined – was a different omega value fit to each time-step? Additionally, why didn't you use a procedural rule to determine omega from the data? There are a couple of possibilities that jump to mind that seem principled. One is to use the model evidence of each agent at each time step (for example, see the "Meta Agent" in Franklin and Frank, Plos Comp Bio 2018). A second possibility is to have another agent that uses prediction error to learn which model should be active at each time step. I can imagine a justification for a strictly observational model here, but I think that justification should be made in the paper.

We thank the reviewer's suggestion. Our initial hybrid model was defined as a time series of free parameters which considerably increased the complexity of the model.

We decided to use such an approach to let the weights be defined by the data. Our primary insight from this model was that the model-based weight remains higher for both the training and generalization phases in the Multiple group. In addition, we found that the model-based weight gradually decreased over time in the Single group (Figure S7), suggesting the transition from model-based to model-free control in the absence of training variability. We have now replaced this unconstrained model by an arbitration mechanism that aims to qualitatively capture such patterns while reducing its complexity. This model predicts different dynamics of the model-based weight for the Single and Multiple groups, and captures the above-mentioned patterns from the unconstrained model (Figure S8). However, it was not able to capture the abrupt change in model-based weights from training to generalization (Figure S7). Our proposed arbitration model was able to best describe the data of various participants at the individual level according to AIC and BIC as can be observed in Tables 1, 2 and 4, although it did not provide the best description overall. Previously, we also considered the arbitration mechanism proposed by Lee et al. (2014) which uses the reliability of the model-free and model-based predictions to modulate the contribution of each system. However, when we performed model recovery (Figure S12), we found that this model was not able to recover its data better than the other models. Indeed, around 40% of the time its data was best recovered by the single-weight or two-weight hybrid models. Therefore, we decided not to incorporate it in the manuscript.

While we believe it is important to model the dynamics of the model-based and model-free interaction, the best models in our experiments at the population level assumed fixed weights.

Minor comments:

page 12,

"Here, we performed a two-step analysis where we first compared performance in the generalization phase to performance at the end of the training phase before comparing potential differences in generalization between the groups"

I found this language to be very confusing.

Our first goal was to determine if each group showed a performance decrement when entering the generalization phase. This was determined by comparing the performance at the end of the training phase with the performance in the generalization phase. We then compared the differences in performance between groups. Our reasoning was that just comparing the performance between groups wouldn't indicate whether either group showed generalization or a lack thereof.

We have now changed the way we describe these analyses (Lines 209-218):

"Of more importance is how well the groups perform when new start-target pairs are introduced in the generalization phase. This was determined by quantifying any potential change in performance between the end of the training phase and beginning of the generalization phase. For the Single group, we found that performance was significantly worse at the onset of the generalization phase compared to the end of the training phase ($t(15) = -5.67$, $p < 0.001$; Figure 2D). In contrast, the Multiple group's performance during generalization was similar to the end of the training phase ($t(15) = -1.21$, $p = 0.242$). When comparing between the groups, it was clear that the Multiple group performed significantly better than the Single group early in generalization (first bin; $t(29.99) = -5.1$, $p < 0.001$; Figure 2C) and still marginally better at the end of the generalization (second bin; $t(29.27) = -1.64$, $p = 0.05$). This change in performance between the groups was significantly different ($t(29.34) = -3.5$, $p = 0.001$; Figure 2D)."

page 12

"For the Single group, we found that performance was significantly worse at the onset of the generalization phase compared to the end of the training phase ($t(15) = -1.21$; $p < 0.001$). In contrast, the Multiple group's performance during generalization was similar to the end of the training phase ($t(15) = -1.21$; $p = 0.242$)."

Is the first t-statistic correct? It looks an error — should be closer to -4

Thank you for pointing it out. Indeed the t-value is -5.67. We have corrected the statistic (Line 212):

"*Furthermore, for the Single group, we found that performance was significantly worse at the onset of the generalization phase compared to the end of the training phase ($t(15) = -5.67$, $p < 0.001$; Figure 2D)"*

page 12

"When comparing between the groups, it was clear that the Multiple group performed significantly better than the Single group ($t(29.9) = -5.1$; $p < 0.001$; Figure 2D), even when controlling for multiple comparisons ($t(29.34) = -3.5$; $p = 0.001$; Figure 2E)."

This reference to Figure 2E looks like a mistake. Figure 2E is RT data

We have corrected the Figure reference and the wording of the sentence (Line 216):

"In addition, the change in performance between the groups was significantly different ($t(29.34) = -3.5$, $p = 0.001$; Figure 2D)."

pg13

"We also examined how planning (reaction time, RT) evolved over training.

RTs were overall higher in the Multiple group group throughout training (Figure 2D; t(29.99) = -4.82; p < 0.001)"

Should be figure 2d

Thank you. It actually refers to Figure 2E, we have corrected it in the manuscript (Line 2020)

pg 13

"Overall, greater generalization in the Multiple group together with higher RTs suggest that this group could have relied more heavily on model-based planning computations involving the visuomotor mapping of the task as opposed to the Single group which could have developed less computationally demanding state-action associations."

This is a pretty big leap from the data — I really don't see how you can reach this conclusion from the RT data presented.

We agree with the reviewer that RTs alone would not be conclusive of the cognitive process generating the data. We have interpreted the higher RT data along with the better generalization performance in the Multiple group as evidence that they were using a flexible model. We have now provided further evidence in this direction. In Figure 3, we show that the Multiple group makes choices during the training phase that indicate a greater use of model-based computations. Particularly, we found that this group more often selects moves that minimize the true distance to the target even when the cursor moves visually away from the target. Such decisions suggest that subjects make decisions based on an extended horizon, which considers the ultimate goal of their actions even if in the short term they appear counterintuitive. We have modified the text (Lines 300-314):

"The differences in generalization performance between the Single and Multiple groups, which remained marginally significant even by the end of the generalization phase, suggest that the training regimes might have led to different learning representations in the task. While both groups chose highly repetitive trajectories during training, which aligns with the memorization of few solutions, the variability experienced by the Multiple group could have simultaneously allowed the better learning of the visuomotor mapping. Evidence supporting this comes from the more frequent selection of moves that exclusively reduce the model-based distance by the Multiple group. In addition, the greater repetition of the preferred trajectories in the Single group given its unique target, could have facilitated the formation of habitual responses which interfered in the generalization phase. Indeed, supplementary analysis revealed that errors in the generalization phase of the Single group more often began with the same key as the one most frequently used trajectory during the training phase, compared to

the Multiple group (Figure S6). While these differences are numerical due to the limited power resulting from the low error rate in the Multiple group, they suggest that beside a greater representation of the mapping, training variability could have also prevented the interference from habitual responses.*”*

page 18

“However, feedback was available during the generalization phase, allowing the Single group to learn the mapping to recover performance, which is suggested by their elevated RTs during the generalization phase. Here, we sought to test this possibility by not providing feedback during the generalization phase and placing the target only one move away from the starting location (Figure 1B)”

How is this different from examining the first trial as in exp 1?
pg18-19

In experiment 1, the participants had feedback of the cursor during the generalization phase. After the first move, the participants could rely on online “closed-loop” control to get to the cursor, which would look like apparent generalization. In addition, they could potentially learn the mapping during the generalization phase. Experiment 2 was designed to remove these two confounds by removing the cursor feedback entirely and having generalization tested just one move away. Alternatively, we could’ve had the generalization targets be multiple moves away, but if we removed the feedback (to prevent online control) and the participant didn’t execute a perfectly planned out movement, then they could never reach the target. In addition, it aimed to explore whether the lack of training variability could impair even simple decisions that required no planning.

“Notably, the performance of the Single group still remained greater than chance (XX statistical test), which suggests that even though they performed worse than the Multiple group”

The statistic is missing here

Thank you. We have incorporated the statistic ($t(15) = 5.88$, $p < 0.001$) in Line 461.

pg 20

“The differences in RTs increase suggest that the Multiple group could readily use the mapping to arrive at the novel locations without the need to deploy further computational resources, whereas the Single group had to switch from state action associations to learn the mapping.”

I don’t think you can make this inference from the data — this is conjecture.

We have now softened the language (Line 476):

"The differences in RTs increase suggest that the Multiple group did not experience a switch in the algorithm used to solve the task, whereas the Single group likely transitioned to a more computationally demanding algorithm. "

pg 25

"While participants who had full knowledge of the mapping optimally arrived at the target more often on average than participants who had less knowledge, this difference was not significant (Figure S4)."

Can you report the test statistic here?

We have now reported the statistic ($t(18.34) = -1.84$, $p = 0.08$) in Line 794.

Pg 41

It seems like the initial value of alpha in the Dirichlet distribution is a free-parameter, no? My interpretation of this model is that you have implicitly defined a uniform prior over the Dirichlet such that all alpha values are equal to 1. I think this is a very reasonable choice, but I will point out that the model may be sensitive to this choice of prior (higher initial values of alpha should tend to make the model less sensitive to data, for example). I think it is better to be explicit

Thank you for pointing this out. Indeed, we have set the initial values of alpha as [1,1,1]. We have now addressed in the text that the model can be sensitive to this choice (Lines 259-261):

> *"Making the initial parameters equal to 1 gives no preference for any direction a priori. While this is a reasonable assumption, the specification of the initial values of the parameters can affect the sensitivity of the model to the data. For example, larger values can make the model less sensitive to data"*

Reviewer #3: Summary:

Laboratory model paradigms for sequential skill learning have used predominantly externally-cued learning but recently efforts have been initiated into exploring internally guided sequence learning, including the current manuscript. In the current work, a variant of Grid Sailing Task (GST) is used for exploring the impact of training variability on the generalization performance on sequences learned de novo.

The control condition in several of these experiments is learning a trajectory that uses single start-goal pair (SSG) and compared with learning trajectories for multiple start-goal pairs (MSG). Novel ideas explored are: introducing stochasticity in the key mappings while participants learnt S-G pairs (in Experiment 4) that discourages trajectory memorization and the use of one-hop test pairs (Exp 2, 3, 4) that obviates the need for planning a trajectory during testing. The other commendable feature is the judicious use of computational models using reinforcement learning (RL) to give a functional account of the plausible processes underlying learning internally-guided discrete sequential skills. Together, the empirical and computational investigations explore the tension between learning chains of local state-action associations (model free MF) versus learning more generalizable mappings (model based MB) while laying down an overall visuomotor map. Overall, the results seem to support the idea that variability during learning in terms of exposure to multiple start-goal pairs or variability in key mappings (KMs) leads to greater generalizability. The current results add to the growing literature on this phenomenon that is observed in diverse learning scenarios. The writing is lucid and the presentation interweaving between empirical and computational investigations is appealing. The plan of statistical tests, the visualizations, and the explanations are commendable.

We thank the reviewer for the positive feedback.

The following are some concerns that the authors could address to improve the manuscript.

Comments (Experimental part):
1) Is the comparison between SSG and MSG conditions fair? Although both conditions (in Exp 1, 2, and 4) have similar number of trials and the goals are all 7 moves away from the start, inherently the experimental conditions do not seem to be balanced. For example, the attentional requirements are different for MSG as compared to SSG (one manifestation of this is visible in the differences in RTs between the two conditions as shown in both Figs 2E and 5E).

> The experimental conditions indeed seem to involve different cognitive demands in participants which are reflected in the differences in their RTs. As specified by the reviewer, by design, the Multiple group faces a more challenging training phase where different start-target pairs are constantly being alternated. While it is common that training variability initially poses greater cognitive demands (Raviv, 2022), we were interested as to whether such training regime would lead to better generalization. We should also note that it is very common to compare learning and generalization between groups that trained with different numbers of targets in studies of sensorimotor learning.

Also, in MSG one has to learn, by design, the key mapping in the face of changing S-G pairs while in SSG, the key mapping needs to be established while the S-G pair remains constant. In this sense, the results do not seem to be surprising -- in the MSG condition,

participants practiced multiple sequences that has likely contributed to their improved performance in generalization. This could be attributed to the mental flexibility developed through practice across various sequences. However, such mental flexibility was not accessible to those in the SSG condition (unless some participants willfully explored several trajectories and have shown better generalization as a result -- perhaps the few outliers in Fig 2C and 5C.

While it is true that the Multiple condition requires participants to have more flexibility in their plan, which could increase some general mental flexibility in planning, we should note that the participants in the Single condition weren't experimentally forced to use a single path. There are multiple optimal paths to the target and, indeed, participants in the Single condition did explore these paths early on in training before settling into a single path (this was in part inspiration for experiment 3). Furthermore, in Experiment 2, we removed the need for more complex planning in the generalization phase by making the target just one move away. To our surprise, the Single group was still significantly worse throughout the generalization phase, suggesting that mental flexibility in planning isn't the entire story. We have incorporated this point in Line 438 of the manuscript:

"Removing the sequential component from the generalization phase can also rule out the potential confound that differences in performance arose due to distinct capabilities to plan new movement sequences resulting from the training regime. Finally, placing the target one step away, removes the possibility that better generalization performance occurs due to greater similarity between the sequences generated during training and the ones required in generalization, as the Multiple group most likely sampled a larger number of sequences."

2) What was the rationale for selecting 260 training trials? Compared to other grid-navigation settings in sequencing literature, the experiments here have a substantially longer training phase. This might potentially lead to over-reliance on instance-based/trajectory-specific learning as opposed to learning generalizable keymaps. This overtraining could lead to pronounced 'habitual' behaviors, especially in the SSG condition thereby amplifying the performance differences with the MSG condition. Since the learning curves show plateau effect around 100-150 trials, would we expect the same results, if the total number of trials were restricted to, say 150?

We chose the number of trials so the experiment lasted between 40-60 min, which corresponds to one hour of credit for our subjects (Princeton students). However, we were able to replicate our findings of Experiment 1 with an online study where we reduced the number of trials to 100 (80 training and 20 generalization).

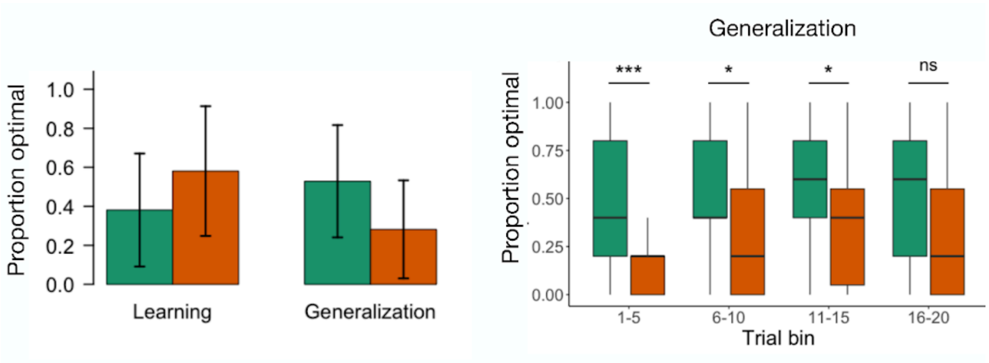

While we do not incorporate these results into the main manuscript, they suggest that habitual responses form rapidly

3) A related question is the variability observed in SSG around 150 trials both in optimal arrivals and RT (Fig 2) -- is this because of fatigue, lapses of attention due to overconfidence of executing overlearned sequence?

While there seems to be a numerical difference in performance in the trials pointed out by the reviewer, we did not find any statistical difference when comparing it with the performance prior or after the referred period in optimal arrivals or RTs.

4) Why is the variability in RT graphs so different for SSG during the learning period between Exp-1 and Exp-2 (Figs 2E and 5E)? Isn't this period identical in the two experiments?

In the following figure we show the RTs from Experiment 1 and Experiment 2 in the Single and Multiple group, and compare them over time using a mixed effect model with time and experiment number as fixed effects and subjects as random effect.

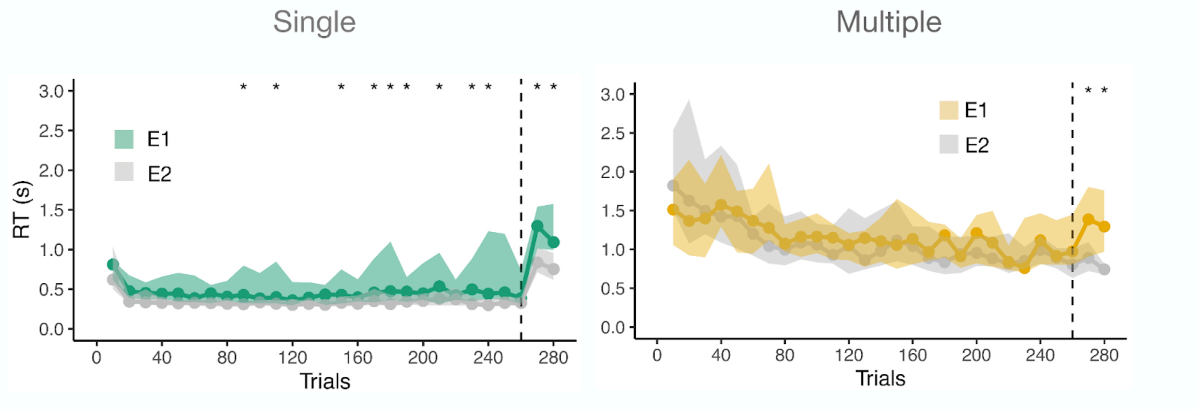

For the Single group we did observe statistical differences ($p < 0.05$) in 9 out of the 26 bins of the training phase primarily in the second half of the experiment. We do not have any explanation for these differences between experiments. As the reviewer suggests, it could be just more attentional lapses in experiment 1 by sampling chance. However, for most of the trial bins, the performance of the Single group in Experiment 1 and Experiment 2 is statistically the same. The difference in RTs in the generalization phase

were expected due to planning given that subjects in Experiment 1 attempted to arrive at distal targets whereas participants in Experiment 2 at proximal targets. For the Multiple group, there were no differences in RTs during training between Experiment 1 and Experiment 2, but there were statistical differences in the generalization phase. As in the Single group, we attribute such differences to planning. We address the latter point in Figure 7 of the main text.

5) In MSG condition, there were 4 different S-G sequences. How do these sequences differ from the ones used in SSG condition, in terms of the complexity of trajectories etc.

Importantly, for the Single group, in order to arrive at the target location the three keys needed to be used, which could suggest that their sequences are more complex in terms of action selection. In contrast, for the Multiple group only two keys were needed to arrive at a given target, although all keys were required across the experiment.

However, another way to determine the complexity of the trajectories is by computing the probability that they occur by chance, with more complex sequences being less likely to be generated randomly. We performed simulations using dynamic programming to compute this probability and found that for the Single group, there was a probability of around 0.06 that a random sequence of seven moves would reach the target. In contrast, for the Multiple group, this probability was around 0.01 for all the start-target pairs. Using this approach, we can say the solution space in the Multiple group is smaller, making the trajectories more complex.

In the answer to a question from the reviewer (below), we present the results of the trajectory analysis used which can provide further insight about their complexity.

Even within the MSG group, is the learning associated with different S-G pairs different?

We did not find that the learning or RTs between the different start-target pairs in the Multiple condition were different. Here is the data for Experiment 1 and 2:

Experiment 1:

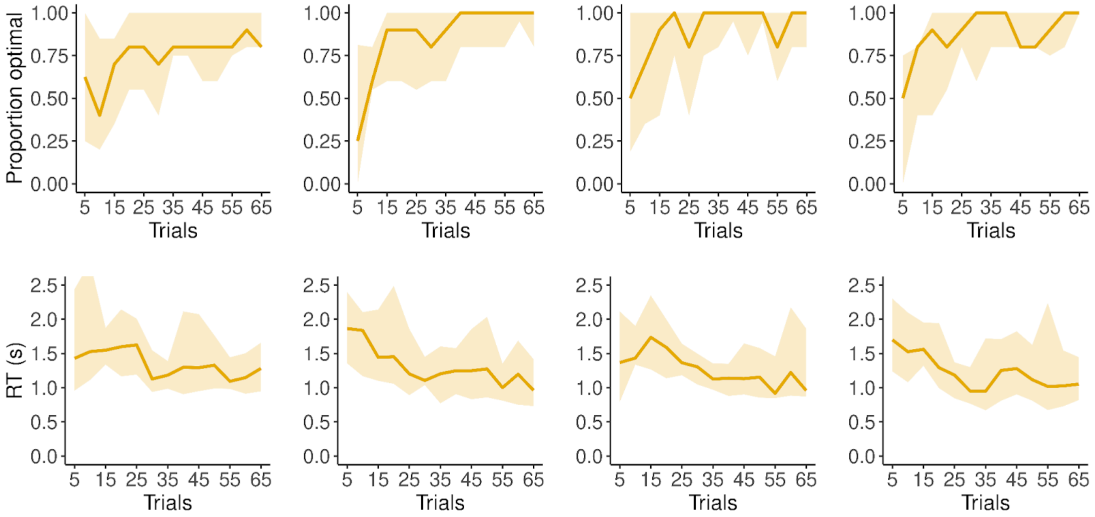

Experiment 2:

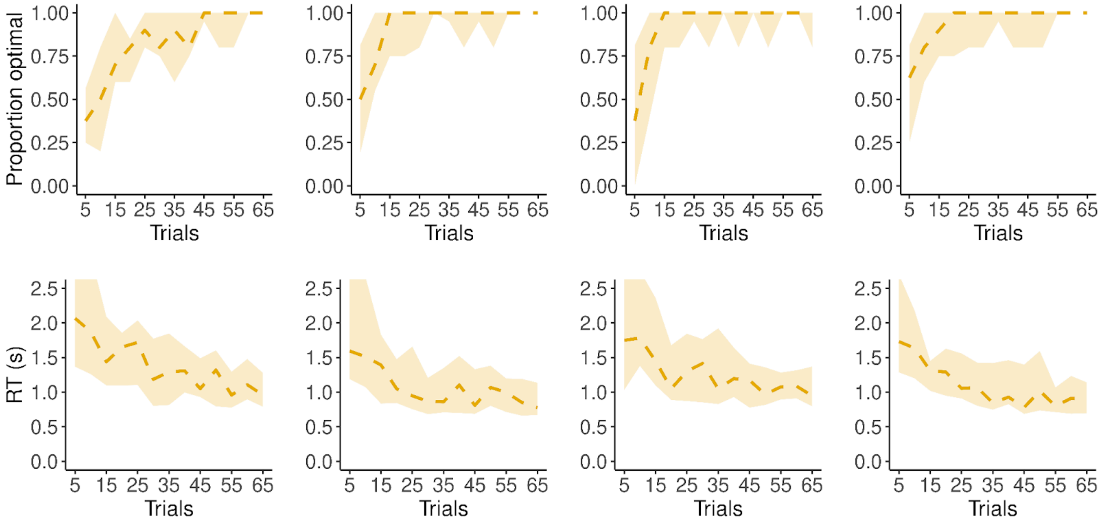

We have incorporated these figures into the Supplementary material (Figure S1 and Figure S9)

One point to consider perhaps is that in SSG example in Fig 1, one solution requires predominant use of J and L and a sparse use of K mapping whereas the sequences for multiple S-G pairs shown in Fig 1 seem to require different (more uniform) pattern of usage of J, K, and L mappings. Do these differences bias the generalization performance?

This is an interesting possibility that could affect generalization performance. One way to answer this is to determine if generalization errors in the Single group were more frequent for the key moving the cursor to the right, which by design was required to be pressed less times to arrive at the target. We focused on generalization trials of Experiment 2 as they require a single move and directly tackles this question. We found that only 17% of the errors in generalization across all participants were related to this key. Therefore, lower generalization performance could not be attributed to the fewer samples the Single group had from the right key.

6) The bin size for visualization as well as statistical testing is chosen as 10 trials. While this might be good for training period, isn't this too small for generalization period where you get only two bins? Does varying bin size to, say 5, show different pattern of results, especially in the generalization results of optimal arrival data (Figs 2C, 2G; Figs 5C, 5G)?

Given that we are reporting the proportion of optimal arrivals (0's and 1's), having bin sizes that are too small would result in more coarse values of performance which would make the variance unstable (e.g., a bin size of 2 would only include values of 0, 0.5 and 1). We thought a bin size of 10 would show more granular values of performance while still showing the variance across participants. However, we found the same behavioral results for bin size = 5:

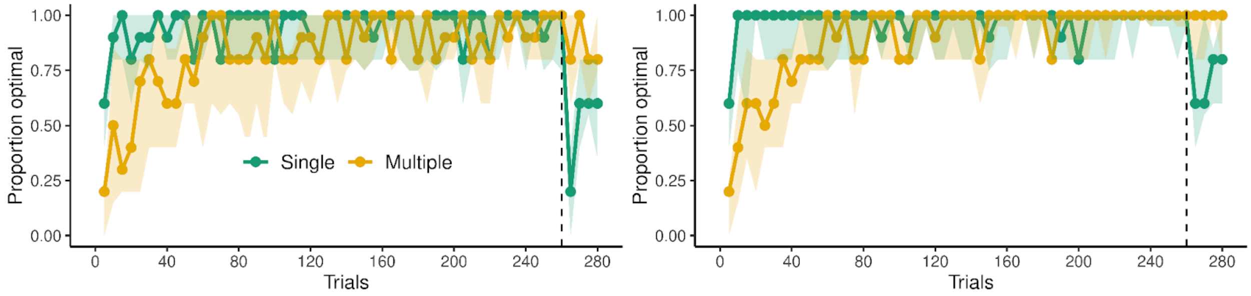

We found similar results to the ones reported in the manuscript. In particular, for Experiment 1, the Multiple group performed significantly better than the Single group in the first ($p < 0.001$), second ($p = 0.01$) and forth ($p = 0.03$) bins of the generalization phase, but not in the third bin ($p = 0.31$). In Experiment 2, the performance of the Multiple group was significantly higher on every bin of the generalization phase (first bin: $p < 0.001$; second bin: $p = 0.001$; third bin: $p = 0.001$; fourth bin: $p = 0.005$). These results corroborate that performance in the single group is significantly impaired compared to the multiple group in the generalization phase.

7) For the generalization phase in Experiments 1 and 2, it would be worthwhile to analyze later generalization trials and not only the initial bin of trials. The performance deterioration of the Single group could also be due to adaptation since they were not

accustomed to multiple S-G pairs during the training phase. This might also partially explain a peak in RT for the Single group in the early generalization phase. While we agree with the authors that the performance on late-phase generalization trials might be confounded with new learning, the slopes of proportion optimal arrivals and execution/reaction time can be analyzed to explore if there are any differences with respect to the initial training phase.

For Experiment 1, even when we did not control for new learning, generalization performance was still marginally lower by the end of the generalization phase (second bin; $t(29.27) = -1.64$, $p = 0.05$). In Experiment 2, when we controlled for new learning, generalization performance was significantly lower at the end of the generalization phase in the Single group (second bin; $t(19.41) = -3.1$, $p = 0.005$).

These results are reported in Line 460.

8) For RT plots (Figs 2, 5), it might help to look at RTs after controlling for variability in the number of moves. This should help in visualizing RT improvements from trajectory-specific motor learning as opposed to that from optimizing trajectory traversals.

We have performed this analysis in point number 5 (above).

Similarly, inter-key interval and execution time could be analyzed while controlling for the number of moves. A significant difference between Single and Multiple groups in inter-key interval trends during the generalization phase would suggest that the Single group needs revisiting KM and subsequent planning to generalize trajectory-independent learning.

We thank the reviewer for this suggestion and we have carried out this analysis with the inter-key-intervals in the generalization phase of Experiment 1. We found that, even though the groups were not different in RTs, they differ in the following 6 inter-key-intervals. This suggests that, as the reviewer pointed out, participants in the Single group had to revisit the mapping to perform planning at intermediate steps of the trajectory.

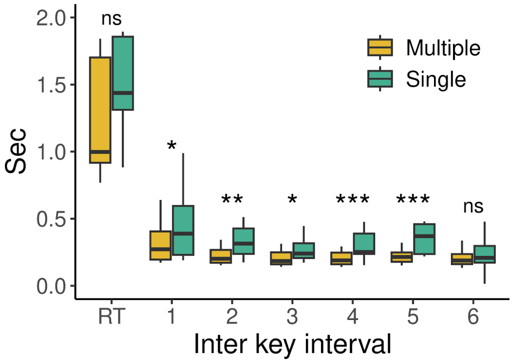

We have added this figure to the supplementary material (Figure S3) and pointed to the results in the text (Line 228).

9) Additionally, trajectories established in Single and Multiple groups could be analyzed. If the keymap was (latently?) acquired during the training phase, we should see a substantial within-subject variation in the trajectories traversed during the training phase.

We thank the reviewer for suggesting the analysis which provided further insights about the heterogeneity of the trajectories used within participants and across participants. In the following figure, we show the most common trajectories traversed by participants in Experiment 1 and 2, for Single and Multiple groups.

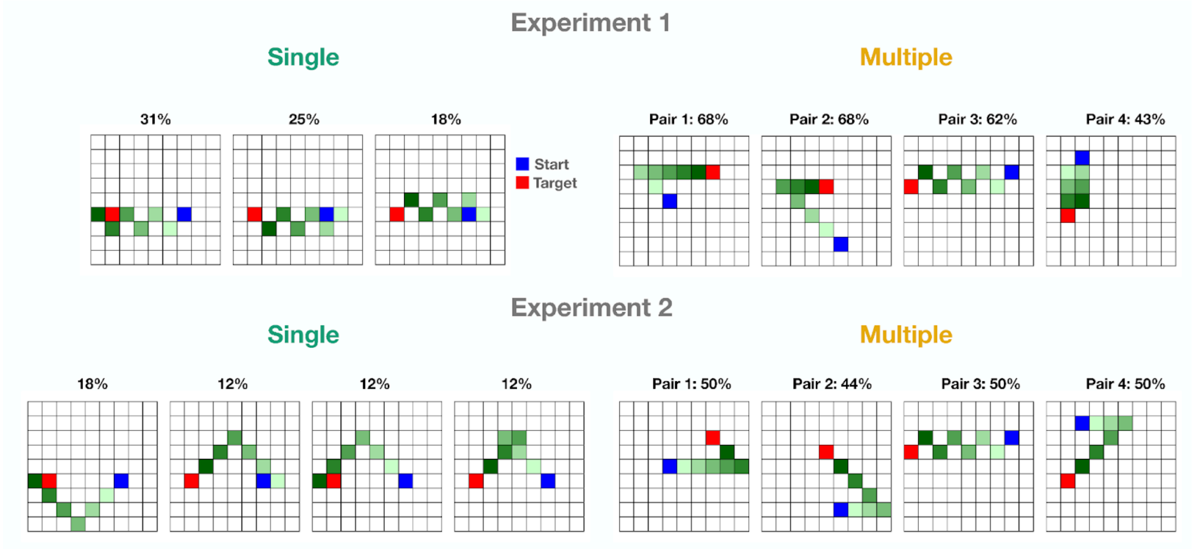

Above each grid, we show the percentage of participants for which the depicted trajectory was the most frequently used during training. We only show trajectories that were most frequently used for at least two subjects. Interestingly, across participants in the Single group, there is a greater heterogeneity in the trajectories used. In particular, for Experiment 1, only 18-31% of participants tended to choose the same trajectory

during training and around 12-18% in Experiment 2. In contrast, around 43-68% of participants in the Multiple group chose the same trajectory for a given start-target pair in Experiment 1, and between 44-50% in Experiment 2. The greater heterogeneity in the trajectories used by the Single group likely resulted from the requirement to utilize all three keys to arrive at the target. In contrast, the Multiple group only required the use of two keys, although all keys were needed across the experiment. Using dynamic programming we found that there were 140 trajectories that optimally arrived at the target in the Single group, compared to 21-35 trajectories in the Multiple group. The greater number of potential trajectories made it less likely that participants would have found the same solutions.

In a related analysis, we found that for a given start-target pair, participants in the Single and Multiple group displayed highly homogeneous trajectories during training:

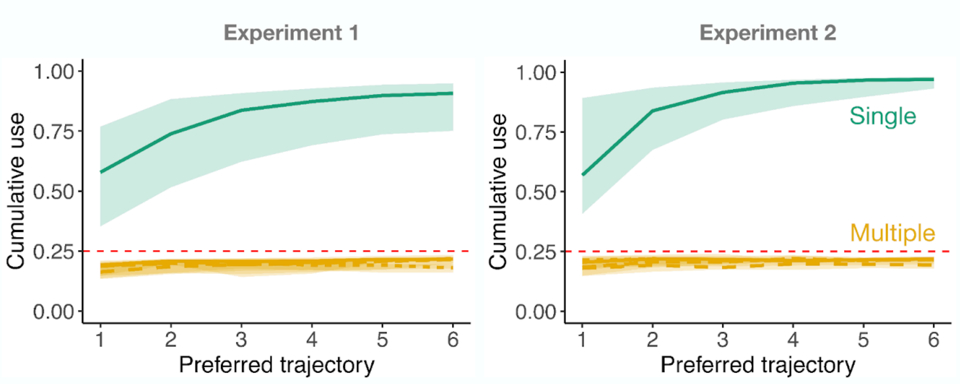

In this figure, we display the cumulative use of the most frequent trajectories during the training phase. The red line indicates the maximum value for the start-target pairs in the Multiple group (proportion = 0.25), given that each pair only appeared in one quarter of the trials. Notably, both groups show a high repetition of the preferred trajectories. In Experiment 1, participants in the Single group reliably chose two trajectories more than 50% of the time (cumulative mean > 0.5, $t(15) = 2.86$, $p = 0.005$). On the other hand, the Multiple group exclusively selected a single trajectory more than 50% of the time on each of the four start-target pairs (cumulative mean > 0.125, $t(15) = [3.28, 2.56, 2.54, 2.05]$, $p = [0.002, 0.01, 0.01, 0.02]$). In Experiment 2, participants in the Single group primarily chose a single trajectory more than 50% of the time (cumulative mean > 0.5, $t(15) = 2.06$, $p = 0.02$). The Multiple group selected a single trajectory more than 50% of the time (cumulative mean > 0.125, $t(15) = [6.21, 6.23, 5.63, 3.91]$, $p < 0.001$). While both groups exclusively chose one or two trajectories relative to their target, the absolute frequency of repetition of the preferred trajectories was smaller in the Multiple group given that, by design, each target appeared for fewer trials. Indeed, the most frequently used trajectory in the Single group was used significantly more times than the maximum number of times the trajectory in the Multiple group could have been used (i.e., 25% of the time; Experiment 1: $t(15) = 4.36$, $p < 0.001$; Experiment 2: $t(15) = 5.88$, $p < 0.001$). The difference in the absolute number of repetitions between the groups most likely contributed to the difference in generalization performance, even if relative to the number of start-target presentations, the Multiple group showed highly repetitive behavior.

Due to the already lengthy manuscript, we have incorporated the previous two figures into the Supplementary material (Figure S4 and Figure S5) and briefly made reference to them in the text (Lines 277, 292, 534, 536) in the behavioral results of Experiment 1 and Experiment 2.

10) Can the results of Exp-4 be motivated and interpreted differently? The knowledge of the keymap is always explicit since the learning in GST is always explicit. It is just that the stochasticity in Exp-4 pushed the participants to learn the KM instead of relying on direct memorization of the trajectories in the Single condition. And that is why you find similar extents of explicit knowledge in Single and Multiple groups. Given this, is it possible to motivate and re-interpret the findings from Experiment 4 in these terms instead of implicit-explicit differences as invoked in the Discussion.

We agree with your excellent interpretation. We have updated the Discussion, where we report that there were no differences in explicit knowledge about the mapping between the groups. As a result, the most likely explanation for the improvement in generalization was that the stochasticity prevented the repetition of memorized responses (Line 939):

> *"Relatedly, when presented with stochasticity in the visuomotor mapping (Experiment 4), which in principle prevented participants from repeating the same sequence of key presses during training, the Single group matched the performance of the Multiple group in generalization. This type of training could have disrupted the formation of habitual responses and allow the flexible use of the learned mapping."*

Also in Lines 1038-1048:

> *"In Experiment 4, we showed that variability in the form of a stochastic mapping can lead to comparable generalization performance between the Single and Multiple groups. However, the significantly lower RTs during the training phase by the Single group, indicate that the improvements in generalization do not come at the expense of higher RTs during training. Therefore, a stochastic training with a single goal, can prove to be an effective regime that maintains the benefits of both model-free and model-based computations. In addition, while we were not able to fully remove the awareness of the mapping in the task, we did not find evidence that differences in awareness between participants were related to generalization performance. We believe further investigation is necessary to test the role of explicit processes in the learning of visuomotor mappings as previous research suggests they might be crucial in tasks similar to ours (McDougle et al., 2022)."*

Comments (Computational part):

11) The state representation used for the grid states is unclear. It is not clear whether absolute positions are used to create the state representation or whether relative positions with respect to the current state and target is used. This is especially important in the case of model-free generalization.

The state representation in our task is in absolute coordinates. We have now made it explicit in the text (Lines 1210).

12) The computational models could have employed sparse rewards i.e., only rewarding the agent when the target was reached as is the case in behavioral experiments. The computational models employ step-by-step reward feedback which seems to be incompatible with the experiment. Moreover, the step-wise reward value for model-free and model-based agents were different which is difficult to justify in a cognitively-grounded view.

Thank you for the suggestion, we have used the chessboard distance as rewards to aid learning in the model because we found learning to be incredibly slow when using sparse rewards. For example, when we implemented a variation of the SARSA algorithm that relies on sparse rewards, we found that it performs remarkably worse than the model that uses the distance reduction as reward. In particular:

Experiment 1: mean Δ BIC = -911 for the Single group and mean Δ BIC = -1644 for the Multiple group.

Experiment 2: mean Δ BIC = -924 for the Single group and mean Δ BIC = -1541 for the Multiple group).

Regarding the difference in the reward signal in the model-free and model-based algorithm. We wanted to make explicit that the model-free algorithm did not have access to the key-mapping knowledge, therefore it could not compute the actual distance to the target in terms of number of moves, but instead it would use a heuristic based on the chessboard distance. On the other hand, we hypothesized that the actual distance to the target (weighted by the uncertainty of the mapping) could be used as a reward in the model-based algorithm as it is meant to represent the internal model. However, we agree with the reviewer that this is not a common assumption, and typically the same reward signal is used. One variation of the BFS algorithm that can address this concern is

13) The hybrid model, especially the relative weighting scheme, could be described in more detail. It is stated that the relative weights were a time series of free parameters (page 32). If this is to mean that the weights used for each trial are completely free, this needs to be justified with a citation. Would it be unfair to compare such models with pure model-free and pure model-based RL algorithms. For example, if each step in a particular trial is predicted accurately by a model-based algorithm, then when fitting for that trial,

the weight can simply be set to maximize the contribution of the model-based algorithms. This is more like switching between model-based and model-free algorithms rather than using a combined hybrid representation. It is also not clear if the weights assigned to a trial remain constant for all steps in the trial. It is interesting to note that the first keypress has a significantly higher reaction time than subsequent keypresses, which indicates more planning early during each trial.
Overall, since the hybrid model could be overparameterized, the set of candidate models could be expanded to include hybrid models without a trial-wise weight as a free parameter.

We have removed the hybrid model with a time series of free parameters from the manuscript and incorporated it into the supplementary materials. Initially, we proposed that model to allow the data to determine the value of the weights, however, this came at the expense of complexity. In the current manuscript, we have incorporated an arbitration mechanism that aims to qualitatively capture the behavior of the weights in the Single and Multiple groups: In particular, these weights remained consistently higher for the Multiple group, decreased over time in the Single group, and increase from training to generalization in both the Single and Multiple group. Our model was able to capture the first two patterns and provided the best description of the data for several subjects in Experiment 1, 2 and 4, although it did not provide the best description at the population level.

The arbitration model, unlike the unconstrained model, provides an update of the model-based weight at every move, which allows for a more granular analysis of the interaction between the systems within a trial.

14) The computational models could benefit from introducing a perseveration parameter and a uniform noise/attention-lapse mechanism. The goodness of fit can then be compared and interpreted.

Thank you for pointing this out. We have started using a perseveration parameter based on Miller et al., 2018 in our follow-up studies to capture participants' convergence on a particular trajectory over training. We haven't considered a uniform noise/attention-lapse mechanism. While the addition of these parameters will likely improve the fit, we are hesitant to add three new models (perseveration, attention-lapse, perseveration and attention-lapse) to the modeling analysis for just an improvement in fit at the cost of an even lengthier manuscript. Instead, we have mentioned this point in the discussion and highlighted it as a limitation of our modeling work (Line 956).

15) Plots for posterior predictives can be included for computational model fits to visualize how the best-fit parameters capture the empirical data.

Given the recursive structure of our new arbitration model to compute the model-based weight, it was not efficient to continue using Gibbs sampling to estimate the distribution of the parameters – therefore we do not provide model checks based on the posterior predictive distribution. We used BADS (Bayesian Adaptive Direct Search, Acerbi, 2020) which instead returns point estimates of the parameters. However, we are evaluating the models in different ways. First, we provide simulations, which align with different guidelines on computational modeling (Palminteri, 2017; Wilson and Collins, 2019) and show that the hybrid model can capture a wide range of generalization patterns (Figure 4). Second, we compare our model with a theoretical near upper bound (Shen and Ma, 2016), which provides a quantitative measure of how well the models capture the empirical data.

16) Justification is unclear for the specific model-based algorithm used. The Grid Sailing Task as given in Fermin, Doya et al. (2010), can be divided into 3 phases: Random (Exploration), Knowledge (Exploitation), and Sequence Memory stages. In the current case, since the keymap is unknown, the participants start from the Exploration stage. This is represented in the model by the initial keymap probabilities in the model being assigned to a uniform Dirichlet distribution (the model does not know what direction the cursor will move for any key press). This probabilistic distribution is then updated using Bayes rule and then used along with an infinite depth BFS algorithm to generate the model-based action probabilities. Notably, such a model is not used in Fermin, Doya et al. (2010) or Fermin, Doya et al. (2016), and is quite different from the suggestions in the 2010 paper.

We generally agree with the stages proposed in Fermin et al. (2010) which resemble the stages of the automatization of a skill (Logan, 1988). We believe more complex tasks are needed in order to study with more detail such stages. In simple grid navigation tasks, like ours, the learning curves saturate quickly and repetitive patterns start to emerge. Suggesting a quick transition from the first to the second and third stages. Currently, we are addressing this point in our lab by asking participants to use more complex mappings (Velazquez and Taylor, 2023). Those tasks represent a richer testbed for the exploratory and exploitative stages, as the sequence memory stage might take hours if not days to occur. However, the models as we have presented them now, contain elements that work as a starting point to formalize the stages. For example, exploratory behavior can be generated at the beginning of the experiment due to the flat prior of the mapping and the absence of state-action values. Exploitation, on the other hand, can occur once the distribution of the mapping has enough information to generate reliable predictions, Finally, the Sequence memory stage can result from a transition from model-based to model-free control. The latter part, as suggested by the reviewer before, can be greatly improved by incorporating a persistence component. In summary, future work can formalize the proposed stages in Fernin et al. (2010) in a hybrid model that incorporates:

- Explorative mechanism (which could be random or directed)
- A biologically plausible algorithm for planning (exploitation stage).

- A persistent component (sequence memory stage)

Editorial Suggestions:

i) References need careful attention:
Pg 9: Dundon et al. (2022)?
Pg 10: Raviv et al. (2022)?
Pg 15: Stephan et al. (2009)
Pg 28: Khan (2018)?
Pg 32: Miller et al. (2019)?

We have now pointed to the references correctly.

ii) In the introduction, the term "control policy" is used in two different contexts - in motor control context and key-map learning context.

We have removed the reference of control policy in the context of the key-direction rule and just refer to it as mapping or visuomotor mapping to avoid confusion with control policy in the motor control literature.

iii) Pg 12 & 13: Figure references seem misplaced at the end of the page -- should these be: Figure 2C and 2D, in place of 2D and 2E? Similarly, 2E and 2H in place of 2D and 2G (top of page 13)?

We have corrected this typo.

iv) Pg 13, Fig 2: The outliers in each group would be interesting to analyze further (Single doing better and Multiple doing worse).

Based on the analysis of the last 25% of the training trials (65 trials), we found two outliers (lower than 1.5 times the interquartile range) in the Single group with an average of 0.48 and 0.55 of optimal arrivals. Also two outliers were found in the Multiple group with 0.6 and 0.3 proportion of arrivals in that period. Performance differences can be attributed to lack of engagement in the task, greater attentional lapses or slower learning rates which we did not investigate further in the paper due to our small sample size per group. However, we did not observe that their lower performance during training was related to their performance in generalization.

For the generalization phase, there was one subject in the Single condition who performed remarkably well (0.9 optimal arrivals). On the other hand, one participant in the Multiple condition performed poorly (0.3 optimal arrivals). The good-performing subject in the Single group was also the one with the lowest RTs in the generalization

phase, indicating a great proficiency in the task despite the not favorable training regime. On the other hand, the participant in the Multiple group was also the one with the highest RTs in the generalization phases, suggesting that the participant was likely getting distracted or the task was unusually difficult for them.

v) Pg 17 "De novo model based learning ...": This is at odds with what is mentioned in methods section (page 35, before Exp 2) -- no feedback given during generalization trials. It needs to be clarified in the Methods section that during the 20 interleaved training trials in the generalization phase, detailed move-by-move feedback was given.

Thank you, we have specified that move-by-move feedback was given on those trials (Line 1131).

vi) Pg 18 last sentence: (XX statistical test) -- which test?

We have reported the statistic ($t(15) = 5.88$, $p < 0.001$) in Line 461.

vii) Pg 20: What is the reason for the differences (in number of LML wins) between Tables 1 and 2, given that the training phase is identical in both -- the best model LML win proportions changed and profiles in Fig 6 are different from Fig 5? Is this because the models now have to account for one-hop generalization instead of multi-hop setting in Exp1 -- but these are relatively few trials as compared to long training?

Differences were likely due to the distinct generalization phases in the experiments. In addition, as mentioned in the text, Experiment 1 contained 20 training trials interleaved with the generalization ones, which could have contributed to this difference. Given that we changed our method of parameter estimation from Gibbs sampling to as our arbitration model was computationally easier to implement this way, we no longer report LML (which was computed with the posterior samples), but now report AIC and BIC.

viii) Pg 27: Figure 9 should be 8?

We have corrected the figure reference.

ix) Pg 35: During the generalization phase, do any of the S-G pairs have partial or full compatibility to any of the training S-G pairs? If so, could these give unfair advantage to Multiple groups?

While the sequences necessary to arrive at the target location in the generalization trials were unique, it was likely that in Experiment 1 the Multiple group could have practiced components of those trajectories during training (we have made reference to this possibility in the text (Lines 976-985). However, in Experiment 2 controls for this potential confound as the targets in generalization require a single move.

x) Pg 35: It might be good to reiterate here in the methods section (also in Fig 1) that the 260 trial training phase is identical in Exp 1 and 2.

We now specify that the training phase in both experiments is the same (Line1143)

xi) Pg 60, Fig S1: Why is the variability for single group inter-key-intervals larger as compared to the multiple group (especially in 1-6)?

We used the Levene's test to assess whether the variance between the Single and Multiple groups was the same for the different inter-key-intervals. We did not find any significant differences in the 1-6 inter-key-intervals.
