# Supplementary material for: The role of training variability for model-based and model-free learning of an arbitrary visuomotor mapping
Source: PLoS Comput Biol. 2024 Sep 27;20(9):e1012471. doi: 10.1371/journal.pcbi.1012471 (PMC11463753; doi:10.1371/journal.pcbi.1012471)
Supplement: Attachment — Submitted filename: Response_Velazquez_Daw_Taylor_R2.docx [file pcbi.1012471.s015.docx]

We sincerely appreciate the reviewers' valuable feedback, which has helped enhance our manuscript. We have carefully addressed all concerns and made the necessary revisions. In addition to these changes, we have included links to the supplementary figures in their captions, now available in the public repository on the Open Science Framework (OSF).

**Reviewer #1: I am satisfied with the authors' revisions and recommend that the work be published. My last lingering concern with the work is minor -- in the authors' response to my questions about experiment 3, they presented a justification for the experiment that did not make the text. In particular, I found the following paragraph helpful to my understanding of their work, and suggest similar language be included:**

**We saw two possibilities, which are outlined in the main text on Lines 611-621: 1) forcing participants to initially learn the mapping may afford generalization even after a long period of repetitive training at a single target (the null hypothesis as the reviewer points out) or 2) participants could eventually forget the mapping if they were only required to repeat movements to one target location. This is like if a budding pianist first learned their scales (“mapping”) but then practiced only a single melody (“sequence”, for an extended period, they might forget the meaning of the piano keys in relation to the scale degrees – the senior author has experienced this firsthand. In addition, in Experiments 1 and 2, it was unclear if the participants in the Single group ever learned the mapping since the task didn’t necessarily demand it. Instead, they could simply memorize a sequence of responses and repeat it throughout the entire experiment. With these two points in mind, we designed Experiment 3 to test whether repetitive training to a single target would afford generalization even after a long period of training or it would cause forgetting of (or interference with) the full mapping, while also ensuring that the participants had learned the mapping in the first place.**

We appreciate the valuable feedback from the reviewer. We have included the paragraph above into the description of Experiment 3 (Lines 599-608):

*“In the previous experiments, we demonstrated that the Single group's performance consistently decreased during the generalization phase. In this study, we investigated whether a short exposure to training variability could prevent this decline, even when followed by a prolonged period with no variability. Encouraging participants to initially learn the mapping may afford generalization even after a long period of repetitive training at a single target. Alternatively, participants could eventually forget the mapping if they were only required to repeat movements to one target location. This is like if a budding pianist first learned their scales (“mapping”) but then practiced only a single melody (“sequence”, for an extended period, they might forget the meaning of the piano keys in relation to the scale degrees.With these two points in mind, we designed Experiment 3 to test whether repetitive training to a single target would afford generalization even after a long period of training or it would cause forgetting of (or interference with) the full mapping, while also ensuring that the participants had learned the mapping in the first place.”*

**Reviewer #2: Overall, the rebuttal comments are satisfactory to the most extent. I commend the authors in conducting additional experiments in addressing the comments and clarifying various things where they offer a rebuttal. Here are few suggestions for improvement. I refer to their point numbers for presenting my concerns/suggestions.**

**1. Point 2: “However, we were able to replicate our findings of Experiment 1 with an online study where we reduced the number of trials to 100 (80 training and 20 generalization).” A statement to this effect can be incorporated in the manuscript/supplement to highlight that this protocol used a longer training period than what is typically found in the literature.**

We would like to thank the reviewer for the detailed feedback. We have incorporated that statement into the main manuscript (Lines 1107-1111) and included the results from the online experiment into the supplementary material (Figure S10):

*“We chose the number of trials so the experiment lasted between 40-60 min, which corresponds to one hour of credit for our subjects (undergraduate students). However, we were able to replicate our main findings with an online study where we reduced the number of trials to 100 (80 training and 20 generalization; Figure S10).”*

Figure S10: Replication of Experiment 1 with Amazon Mechanical Turk participants. The Single group is represented in green and the Multiple group is represented in gold. https://osf.io/ds36y

**2. Point 7: The authors misinterpreted the point of this comment. We meant to suggest that SSG group participants are not exposed to the multi-SG condition. Hence, a peak in RT during the generalization period might suggest adaptation to some extent. A potential way to look at this would be to analyze trial-wise comparison of differences in RT during the generalization period.**

Our response to point 7) addressed the reviewer's sentence *“The performance deterioration of the Single group could also be due to adaptation since they were not accustomed to multiple S-G pairs during the training phase”* . Which we believe is true to some extent in the initial trials of generalization, but does not explain that in Experiment 2 the lower performance of the Single group remains until the end of the Experiment. We also make reference to this in the discussion pointing to change-point adaptation experiments (Lines 964-966):

*“In contrast, studies on change-point detection find that performance typically recovers immediately after an abrupt transition when a controller is well known, at least in simple scenarios [83,84].”*

In terms of RTs, Figure 2E does not show significant differences in RT between the groups in the generalization period over trial bins. However, we performed the trial-by-trial analysis suggested by the reviewer and found that in the very first trial the Single group had significantly higher (p<0.05) RT than the Multiple group, which could be attributed to not expecting to see a novel target. However, there are no differences between the groups in the rest of the trials.

**3. Point 8: The authors do not seem to address this comment – point number 5 refers to a different kind of analysis. We intended to suggest looking at the normalized RT plots (RT divided by the number of moves) here to control for the number of moves.**

Thank you for the clarification. We normalized the RT by the number of moves in Experiment 1 and did not find noticeable differences from the 2E plot, beyond rescaling the values. Therefore, we decided not to incorporate these results into the manuscript.

**4. Point 12: While we agree with the authors’ argument that RL models with sparse rewards performed poorly as compared to those with dense rewards, there is a bit of contradiction here in terms of the interpretation. The chessboard distance reward metric used in these models may not exactly correlate with the KM-based distance. The observation made by the authors that sometimes participants ‘seemingly’ move the cursor away from the target in terms of visual distance while still getting closer in terms of the KM distance can be considered as evidence for model-based learning should be interpreted with caution. Moreover, the point that SARSA with sparse rewards performed poorly could be mentioned in the manuscript or supplement.**

We appreciate the suggestion. In the first revised manuscript we included the reference to SARSA both in the main manuscript (Lines 1226-1228) and in the supplementary material (Figure S11).

*“In a supplementary analysis (Figure S11), we show that this model-free algorithm performs better or at the same level than SARSA, a commonly used temporal-difference algorithm [20]. We believe that the reduction in the chessboard distance is an intuitive measure of reward in this model, as it is equivalent to visually getting closer to the target.”*

Figure S11: Model comparison between our model-free algorithm (here MF*) and SARSA (20). SARSA provides a temporal difference update to state-action values for every start-target pair: Q(s,a)←Q(s,a)+α[r+γQ(s′,a′)−Q(s,a)]. We evaluated the models in the data of Experiment 1 and Experiment 2 using AIC and BIC differences and testing if they were different from zero using the Wilcoxon signed-rank test. We found that our model performed the same or better than SARSA depending on the metric. In particular, the AIC difference was significantly in favor of our model for the Single group (V = 34, p = 0.04), although there was no difference between the models in the Multiple group (V = 77, p = 0.33). According to BIC, which penalizes more strongly the extra parameter γ in SARSA, our model-free algorithm was significantly better for the Single (V = 0, p < 0.001) and the Multiple group (V = 26, p = 0.01). We found similar results for the data on Experiment 2, with no significant difference in performance between the models for the Single (V = 46, p = 0.13) or the Multiple group (V = 67, p = 0.97) according to AIC, but our model outperformed SARSA for both the Single (V = 31, p = 0.02) and the Multiple group (V = 26, p = 0.01) according to BIC. https://osf.io/u85ce

**5. Point 13: The authors can potentially include a candidate model that is a parametric arbitration model. This model would introduce a good baseline comparison for how an arbitration model (that calls for a model-free or model-based evaluation at each time step in a binary sense unlike the hybrid model that employs weighted estimates from the two systems) performs when the weights are not defined using a series of free parameters.**

Since the comparison of the models was done using AIC and BIC, the parametric version of the arbitration significantly underperforms the rest of the models due to its many parameters. We believe it would not be very informative to include it in the main results, but we have incorporated the AIC and BIC values and a brief explanation in Figure S7 (Line 1723), where we had plotted the weights of the unconstrained model.

*“Since this model has a free parameter per trial, it underperforms the rest of the models when complexity is measured as the parameter counts (Experiment 1: Single: ΔBIC = 2445, Multiple: ΔBIC = 2280; Experiment 2: Single: ΔBIC = 2457, Multiple: ΔBIC = 2214. ΔBIC is the BIC difference in medians with the best model from Tables 1 and 2). However, it provides valuable insights into the dynamics of the weights, which we use to build the arbitration model (AR).”*

**6. Point 14: Given a substantial amount of variance in RT plots as well as an (in our opinion) unnecessarily long training period, we think it is crucial to introduce perseveration and attention-lapse parameters in the modeling. Without introducing these parameters, our interpretation of best-fit parameters may be conditioned on some model mimicry effects wherein the model may try to adjust the parameters in order to capture the attention lapses and perseveration in the behavioral data.**

While we generally agree with the suggestion of including a perseveration component, we have opted to address it as a limitation in our study. We refer to this point extensively in the Discussion (lines 935-955). As we specify in the text, perseveration can be thought to occur at different levels. However, over the experiment, perseverative responses are also likely to reduce the chessboard distance (our measure of reward). Therefore, further experimental manipulations would be necessary to dissociate them behaviorally and in the models. From the discussion:

*“Previous research has demonstrated that a key characteristic of habitual responses is their persistence after reward devaluation [76,77]. However, a history of reward might not be necessary for the formation of habits as they can emerge simply through the repetition of actions [78,79,80]. While our models implement reward as visually minimizing the (chessboard) distance to the target, it is unclear the extent to which this is actually rewarding for participants. In addition, the explicit presentation of rewards in our task (emoji faces at the end of the training trials) was relatively sparse. Therefore, it seems more plausible that any habitual response emerging during generalization, resulted from its frequent repetition during the training phase [78,79] (Figure S6), rather than due to its strengthening from reward. One major limitation of our work is that the current version of our models do not explicitly account for such autocorrelation of responses. Instead, our implementation of the model-free system only predicts chance-level responses in the face of novel start-target pairs. This assumption is not accurate and would likely benefit from the incorporation of a persistence component of previously taken actions [79,81,82]. The exact level at which persistence can occur in our task is an empirical question. For example, it can manifest at the global level if a subject has a tendency to press one key more often than the others throughout the task, regardless of the start-target pair or the current location of the cursor. Alternatively, persistence can occur at a more local level, where responses would be more likely to be repeated on a given grid state, or for a given start-target pair. Allowing the models to capture habitual responses during generalization could improve the model fits and lead to a more pronounced difference in the model-based weights between the Single and Multiple groups.”*

**7. Point 15: We are not sure what the authors are trying to suggest with “Given the recursive structure of …. It was not efficient to use Gibbs sampling”. A full posterior estimation should be plausible even with the kind of model definitions used in this manuscript. Additionally, even with the point estimates, the models should be simulated with the best-fit parameters and the simulated data should be compared to the empirical data.**

Thank you for the clarification. As the reviewer pointed out, the issue wasn't related to the recursive nature of the models. Specifically, the arbitration model (AR) required tracking previous states and their frequencies, which we found impractical to implement in JAGS due to syntax limitations and excessive computational demands. Consequently, we chose to utilize the more flexible Matlab interface and the faster point estimates provided by BADS.

In terms of how the models describe the empirical data, we do provide a measure of this considering that not all the variability can be explained by the models (Shen and Ma), and therefore they should be compared with upper bounds, like the negative entropy. We believe this provides a more rigorous way to test the absolute goodness of fit of the models.
